# Efficacy and Safety of Orally Administered East Asian Herbal Medicine Combined with Narrowband Ultraviolet B against Psoriasis: A Bayesian Network Meta-Analysis and Network Analysis

**DOI:** 10.3390/nu16162690

**Published:** 2024-08-13

**Authors:** Hee-Geun Jo, Hyehwa Kim, Eunhye Baek, Jihye Seo, Donghun Lee

**Affiliations:** 1 Department of Herbal Pharmacology, College of Korean Medicine, Gachon University, 1342 Seongnamdae-ro, Seongnam-si 13120, Republic of Korea; jho3366@hanmail.net; 2 Naturalis Inc., 6, Daewangpangyo-ro, Bundang-gu, Seongnam-si 13549, Republic of Korea; 3 KC Korean Medicine Hospital 12, Haeol 2-gil, Paju-si 10865, Republic of Korea; 4 RexSoft Inc., 1 Gwanak-ro, Seoul 08826, Republic of Korea; 5 Siho Korean Medicine Clinic, 407, Dongtansillicheon-ro, Hwaseong-si 18484, Republic of Korea

**Keywords:** East Asian herbal medicine, psoriasis, Bayesian network meta-analysis, systematic review, network analysis, NB-UVB

## Abstract

Psoriasis is a chronic, immune-mediated inflammatory skin disease with many complications and a poor prognosis that imposes a significant burden on individuals and society. Narrowband ultraviolet B (NB-UVB) represents a cost-effective non-drug therapeutic intervention for psoriasis. East Asian herbal medicine (EAHM) is currently being investigated for its potential as a safe and effective psoriasis treatment. Consequently, it has the potential to be employed as a combination therapy with NB-UVB. The objective was to ascertain the efficacy and safety of the EAHM with NB-UVB combination therapy and to identify important drugs for further research. In this study, randomized controlled trials (RCTs) were retrieved from ten databases in Korea, China, and Japan. All statistical analyses were conducted using R software version 4.3.0. The primary outcomes were the Psoriasis Area and Severity Index (PASI) and the incidence rate of adverse events (AEs), while the secondary outcomes were hematologic markers and the Dermatology Life Quality Index (DLQI), which reflect the immune-mediated inflammatory pathology of psoriasis. The analysis of 40 RCTs, including 3521 participants, demonstrated that EAHM with NB-UVB combination therapy exhibited a statistically significant superiority over NB-UVB monotherapy with respect to primary and secondary outcomes. The Bayesian network meta-analysis revealed that Investigator Presciption 3 and Ziyin Liangxue Decoction exhibited a consistent relative advantage with respect to each PASI-based efficacy metric. The network analysis estimated the potential influence ranking for all individual herbs according to PageRank centrality. The findings of this study suggest that EAHMs co-administered with NB-UVB may provide additional efficacy and safety-related benefits for patients with psoriasis. However, the quality of evidence is still low, and further high-quality trials are needed to reach more definitive conclusions.

## 1. Introduction

Psoriasis is an immune-mediated inflammatory skin disease characterized by chronic progression and a diverse array of comorbidities [1]. While not investigated in all countries worldwide, the reported prevalence of this condition varies considerably, ranging from 0.14% in East Asia to 1.99% in Australasia [2]. The global incidence of psoriasis has demonstrated a persistent upward trajectory in recent decades. According to available data, the incidence rate of new cases increased from 92 per 100,000 individuals in 1990 to 99 per 100,000 in 2017 [3]. Psoriasis poses a considerable burden on individuals and societies globally, making the development of improved therapeutic approaches a crucial medical research priority. Due to the incompletely elucidated pathogenesis of psoriasis, definitive curative treatments remain elusive. Current therapeutic approaches are broadly categorized into topical treatments, including corticosteroids and vitamin D analogs, phototherapy, oral systemic agents, and biologic therapies [4]. Oral systemic agents, while the longest-established psoriasis treatment, are controversial due to their extensive adverse drug reactions, including hepatotoxicity, nephrotoxicity, hematotoxicity, and carcinogenicity [4,5]. Biologics offer markedly enhanced efficacy and safety profiles; however, their relatively high cost restricts accessibility for numerous patients facing financial constraints [6,7]. Consequently, topical treatments and phototherapy are primarily considered first-line options for patients with psoriasis. Notably, phototherapy offers the advantage of delaying the initiation of drug-based treatments, potentially resulting in substantial healthcare cost reductions [8]. Broadband ultraviolet B (BB-UVB) and narrowband ultraviolet B (NB-UVB) have been the most widely used phototherapies. Recently, NB-UVB has largely supplanted BB-UVB due to its superior efficacy in treating not only mild but also moderate-to-severe generalized psoriasis [9]. NB-UVB, being a non-pharmacological treatment, can be combined with various topical or systemic medications to achieve synergistic effects. Indeed, such combination therapies are recommended in several clinical guidelines [10]. Recent cost-effectiveness studies indicate that while NB-UVB demonstrates economic advantages, its cost-efficiency may be compromised relative to other therapies due to the required frequent clinic visits, potentially affecting patient adherence [11]. From this perspective, there is a continuous need to develop additional combination therapies that significantly enhance NB-UVB’s efficacy while mitigating its potential adverse effects.

East Asian herbal medicine (EAHM) represents one of the most extensively researched subcategories of globally bioactive natural products. “EAHM” refers to a group of medically recognized natural products that have been used to treat hundreds of millions of people since their theoretical foundations in ancient China and have been incorporated into the pharmacopeias of many East Asian countries, including Korea, Japan, Thailand, Vietnam, and Singapore [12,13,14,15]. EAHM, based on its unique theory of personalized compound formulation, is expected to show efficacy in various immune-mediated inflammatory diseases [16,17,18,19,20]. Indeed, an expanding corpus of research is attesting to the efficacy of EAHM in the treatment of psoriasis and elucidating the underlying mechanisms [21,22,23,24,25,26]. Despite the potential of EAHM as a promising adjuvant therapy for NB-UVB treatment, research in this area has remained limited since the publication of a meta-analysis in 2015 [27]. While the aforementioned studies have indicated favorable outcomes when EAHM is employed as a combination therapy, further investigation is required to enhance the robustness of this conclusion in order to provide a more comprehensive understanding of the subject matter. Firstly, the previous systematic review only analyzed 18 randomized clinical trials (RCTs). These RCTs all examined the efficacy of orally administered EAHM and NB-UVB combination therapy for psoriasis, and the existing systematic review synthesizing these data as of 2015 provides important information as a preliminary study for the purpose of this study. However, to address the limitations imposed by the restricted statistical power resulting from the modest sample size, it is essential to incorporate data from recently conducted additional RCTs spanning the past decade into the analysis. Furthermore, as EAHMs are typically administered in a combination of multiple herbs, there is considerable heterogeneity between the individual RCTs. On the basis of the characteristics of this intervention, it seems appropriate to perform a network meta-analysis in addition to a traditional meta-analysis in order to determine the comparative effectiveness of EAHM combination therapies across different RCTs.

In light of the contextual background and prior research, our study was designed to test the hypothesis that EAHM is a suitable candidate pool for combination therapy with NB-UVB, with the aim of enhancing efficacy and safety, and extending the conclusions of previous studies on the subject. A systematic review was initially conducted, which involved a more expansive scope of the search than had been employed in previous studies. The efficacy and safety data collected were subjected to pairwise meta-analysis and Bayesian network meta-analysis in sequence, with the objective of deriving both direct and indirect evidence. In addition, the mathematical relationships between the networks of all individual components of EAHMs within a prescription were analyzed, with a view to deriving an importance ranking for each component based on a centrality metric.

## 2. Materials and Methods

### 2.1. Database and Literature Search Strategy

This systematic review and Bayesian network meta-analysis was conducted in accordance with the reporting guidelines of The PRISMA extension statement for reporting of systematic reviews incorporating network meta-analyses of healthcare interventions [28]. The protocol and amendments of this study were prospectively registered in the PROSPERO database (CRD4202450009). The following 10 databases were searched from inception to October 2023 for randomized controlled trials (RCTs) of the efficacy of EAHM and NB-UVB combination therapy for psoriasis: three English databases (PubMed, Cochrane Library, and Embase), four Korean databases (Korean Studies Information Service System, Research Information Service System, Oriental Medicine Advanced Searching Integrated System, and Korea Citation Index), two Chinese databases (Chinese National Knowledge Infrastructure Database, Wanfang data), and one Japanese database (Citation Information by National Institute of Informatics). The literature search was undertaken independently by two investigators (HGJ and HK). A detailed database-specific search strategy can be found in Supplementary Material S1.

### 2.2. Study Selection Process

For this review, only randomized controlled trials of the efficacy and safety of EAHM with NB-UVB combination therapy were included in the analysis. No language or geographic restrictions were imposed as long as the inclusion criteria were met. We excluded studies that met the following criteria: (1) the intervention in the experimental group was not EAHM with NB-UVB combination therapy; (2) the disease under study was unrelated or unclear to psoriasis vulgaris; (3) the study design did not meet the definition of a randomized controlled trial; (4) the route of administration of EAHM was not oral; (5) the study was a case report, observational study, or review; or (6) the study was not published in a peer reviewed scientific journal.

There were no restrictions on age, gender, or race with respect to the participants. We included only studies in which patients with psoriasis were screened by objective and explicit diagnostic criteria. We excluded studies of disease subtypes other than psoriasis vulgaris, such as psoriatic arthritis, guttate psoriasis, palmoplantar pulposus, and erythrodermic psoriasis. 

For interventions, only RCTs with EAHM with NB-UVB combination therapy in the intervention arm and NB-UVB monotherapy in the control arm were considered. EAHM was limited to studies that were administered orally, and no restrictions were placed on the drug formulation or duration of treatment as long as the above intervention-related criteria were met. However, studies that did not specifically report the individual herbs that constitute the EAHM formulation were excluded from the review. 

The primary outcomes were the Psoriasis Area Severity Index (PASI), a validated instrument that measures the overall skin damage caused by psoriasis across four areas of the body, and the incidence of adverse events (AEs) [29]. Since there is no international consensus on the Minimal Clinically Important Difference in the PASI, this study utilized PASI improvement of 60% (PASI 60), PASI improvement of 90% (PASI 90), and numerical improvement in PASI score as the primary outcome measures. As secondary outcomes, we selected interleukin (IL)-2, IL-8, IL-17, IL-22, and IL-23, interferon gamma (IFN-γ), tumor necrosis factor alpha (TNF-α), and CD4+/CD8+ ratio as indicators reflecting immune-mediated inflammatory pathology control, and the Dermatology Life Quality Index (DLQI) was selected as the indicator reflecting quality of life. 

### 2.3. Data Extraction 

Two researchers, HGJ and HK, conducted a full-text review based on the inclusion and exclusion criteria to select articles for inclusion in this review. The two researchers then independently explored and extracted the following item data for all included individual articles:

(1) Publication information: title, first author, publication year;
(2) Characteristics of study: trial design, sample size, randomization methods, duration of intervention;
(3) Participants characteristics: age, sex, inclusion criteria, number of participants in each group;
(4) Interventions: experimental group intervention, control group intervention, dose and frequency of drug administration, and drug composition;
(5) Outcomes: Primary and secondary evaluation metrics, measurement point, incidence rate, and detailed information of AEs.

### 2.4. Evaluating the Quality of Study

Assessment of individual study quality was performed independently by two investigators (HGJ, HK) utilizing a revised tool for assessing risk of bias in randomized trials (RoB 2) [30]. In addition, in cases of disagreement between two researchers, consensus was reached through discussion moderated by a third author (DL). RoB 2 classifies the methodological quality of individual studies into three categories: “high risk of bias”, “low risk of bias”, and “some concerns”. For each study, the assessment was conducted comprehensively across five domains of quality: randomization process, bias deviating from the intended intervention, bias due to the omission of outcome data, and bias in the selection of reported outcomes.

### 2.5. Quality of Evidence according to Outcome Measures

The quality of direct evidence for each outcome was assessed using the Grading of Recommendations Assessment, Development, and Evaluation (GRADE) pro framework [31]. This framework categorizes the quality of evidence into four levels: very low, low, moderate, and high, which are influenced by the presence of risk of bias, inconsistency, indirectness, imprecision, and publication bias.

### 2.6. Statistical Analysis

All statistical analyses in this study, including pairwise meta-analysis and Bayesian network meta-analysis, were conducted utilizing R software version 4.3.0 (R Core Team (2023). R: A language and environment for statistical computing. R Foundation for Statistical Computing, Vienna, Austria) and R studio program (Version 2023.12.0+369, Posit team (2023). RStudio: Integrated Development Environment for R. Posit Software, PBC, Boston, MA, USA. URL http://www.posit.co/, accessed on 8 August 2024).

#### 2.6.1. Pairwise Meta-Analysis

In this study, pairwise meta-analysis for a direct comparison between EAHM with NB-UVB combination therapy and UVB monotherapy was performed by adjusting the settings in the R package meta for each analysis condition [32]. Effect sizes and 95% confidence intervals (CIs) from the pooled data were estimated with random effect models only, given the strong heterogeneity of EAHMs among component drugs. The effect size indices for categorical data, PASI 90 and PASI 60, were analyzed by relative risk (RR), and the incidence rates of AEs were analyzed by the odds ratio (ORs) because they deal with events with a relatively low probability of occurrence. Mean difference (MD) was used to calculate the effect of numerical data, PASI score, CD4+/CD8+ ratio, and DLQI, and standardized mean difference (SMD) was used for IL-2, IL-17, IL-22, IL-23, IFN-γ, and TNF-α due to differences in reported units between studies. For the estimation of heterogeneity variance, tau squared statistics (*t*^2^) and I squared statistics (*I*^2^) were utilized as estimators. However, in the case of *t*^2^, we adopted the DerSimonian–Laird estimator for continuous outcome data, but we utilized the Paule–Mandel estimator for binary effect size data because it has better properties as an estimate [33]. Baujat plot analysis [34], Graphic Display of Heterogeneity (GOSH) plot analysis [35], and Leave-One-Out sensitivity analysis were all performed for influence analysis if the primary outcome synthesis showed a value of *I*^2^ = 50% or greater. For outcomes with more than 10 studies, we assessed publication bias qualitatively and quantitatively using contour-enhanced funnel plots and Egger’s Regression test [36,37]. If publication bias was detected, we estimated the corrected pooled effect size based on the Duval and Tweedie trim and fill [38].

#### 2.6.2. Network Meta-Analysis

A Bayesian network meta-analysis was conducted on the four outcomes, PASI 90, PASI 60, PASI score, and AEs incidence, for which at least 10 studies reported data. The objective was to explore the ranking of efficacy between different EAHM and UVB combination therapies. For this analysis, the settings of BUGSnet, an R package for Bayesian network meta-analysis, were adjusted for each test condition [39]. For PASI 60 and PASI 90, efficacy was measured as RR with a 95% credible interval (Crl) using the binomial distribution assumption and log link function. For the AEs incidence rate, the results were estimated as OR with 95% Crl based on the binomial distribution assumption and logit link function. For PASI score, a numerical variable, the results were analyzed as MD with 95% Crl by applying a normal likelihood model and identity link function. In the previous pairwise meta-analysis, only the random effect model was used as a data synthesis mode, but in the network meta-analysis, both random- and fixed-effect models were built first, and the better model was selected by evaluating the terms of convergence and fit. The most appropriate model was then selected through a process of mutual comparison between models, using leverage plots and deviance information criterion ($DIC=-2\log\left(y\right|\hat{\theta })+{2p}_{DIC})$ [40]. Markov chain Monte Carlo simulations were employed to extract the value of interest at each tenth interval. The simulations were conducted with a burn-in of 20,000 iterations and a total of 50,000 iterations. The results of the intercomparison were graphically represented as League heatmaps based on the relative effect estimates derived from the network meta-analysis. Furthermore, surface under the cumulative ranking probability (SUCRA) plots were presented in order to rank the interventions. Inconsistency was evaluated by constructing an additional $d_{\left(t_{i1},t_{ik}\right)}\neq d_{\left(1,t_{ik}\right)}-d_{\left(1,t_{i1}\right)}$ model that did not satisfy the transivity assumption and plotting the individual data point-based posterior mean deviance contributions against the consistency model.

#### 2.6.3. Network Analysis of Component Herbs in EAHM Prescriptions

Given that EAHMs are essentially multidrug combination prescriptions, their constituent herbs are highly diverse, and the contribution of individual herbs to their effects may vary, necessitating separate analysis. In this study, the constituent drugs of EAHMs were considered nodes of the network, and PageRank centrality was utilized to calculate the influence of individual herbs on pharmacological effects through network analysis [41]. This centrality index is characterized by the fact that actors that play a key role in connecting other nodes to each other have a higher centrality index, which is suitable for the purpose of the above analysis. The PageRank centrality for an arbitrary herb h can be expressed as follows:

$$PR\left(h\right)=\sum_{h\in P_{h}}\frac{PR(h)}{L(h)}$$

In other words, the PageRank index for herb *h* is expressed as the PageRank value for each herb *h* in the set *P_h_*, which contains all herbs connected to herb *h* within an EAHM prescription, divided by the number of combinations *L(h)* of herb *h* with other drugs.

## 3. Results

### 3.1. Characteristics of the Included Literatures

**Figure 1:**
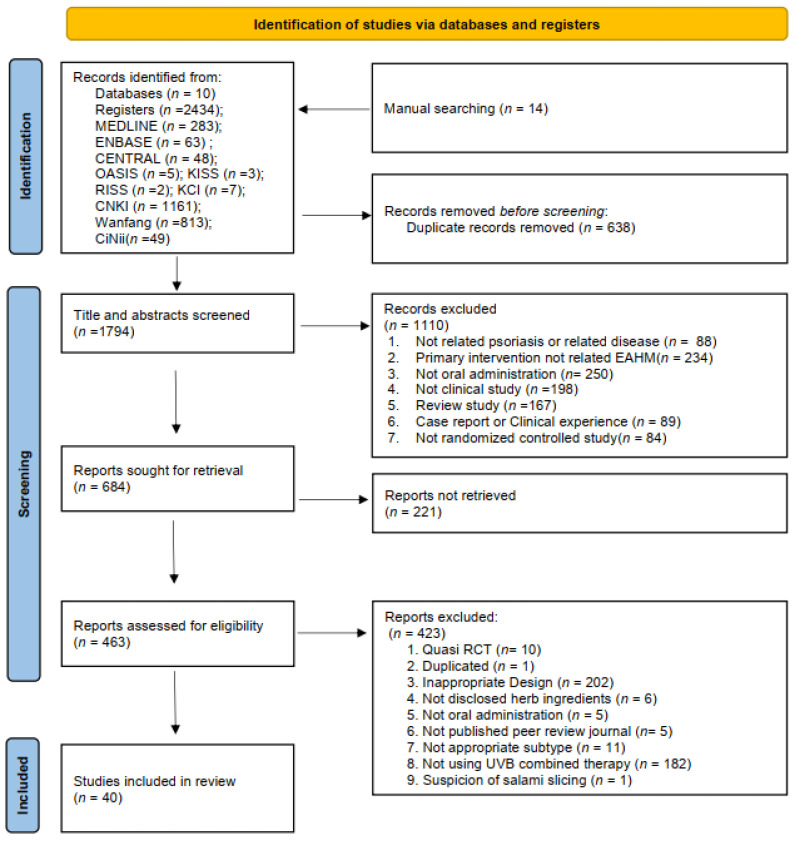
Flow diagram of the literature screening using the PRISMA 2020 format.

A pre-established strategy was employed to retrieve 2434 potentially pertinent articles from 10 databases, with an additional 14 articles identified through manual searching. Following the removal of duplicates, 1794 articles were initially identified. Following a preliminary screening of titles and abstracts, 1110 articles were excluded based on the defined exclusion criteria. Of these, 463 articles were subjected to a full-text evaluation, provided that the full text was available. Finally, 40 articles were selected for inclusion in this systematic review, as they met the objectives of the review. The complete literature selection process is outlined in the PRISMA 2020 flow diagram below (Figure 1).

A summary of the basic information about the 40 included studies can be found in Supplementary Material S2 [42,43,44,45,46,47,48,49,50,51,52,53,54,55,56,57,58,59,60,61,62,63,64,65,66,67,68,69,70,71,72,73,74,75,76,77,78,79,80,81]. The sample size of the individual studies ranged from 51 to 200, with a total number of 3521 participants. The experimental group *n* = 1804 and the control group *n* = 1717. In all 40 studies, the control intervention was NB-UVB monotherapy, while the experimental intervention was EAHM with NB-UVB combination therapy. The EAHM prescriptions that were adopted by each study were Bai Bi Decoction (BBD, *n* = 1), Biejia Jian Pills (BJPs, *n* = 1), Compound Qingdai Capsule (CQC, *n* = 1), Huaixiang Wushe Granule (HWG, *n* = 1), Huoxue Jiedu Decoction (HJD, *n* = 1), Investigator Presciption1 (IP1, *n* = 1), Investigator Presciption2 (IP2, *n* = 1), Investigator Presciption3 (IP3, *n* = 2), Keyin Pill (KP, *n* = 1), Liangxue Jiedu Decoction (LJD, *n* = 1), Liangxue Shufeng Decoction (LSFD, *n* = 1), Liangxue Xiaobian Decoction (LXBD, *n* = 1), Liangxue Xiaofeng Decoction (LXFD, *n* = 2), Liangxue Zhiyang Decoction (LZD, *n* = 1), Modified Liangxue Xiaoyin Decoction (MLXD, *n* = 1), Qingre Liangxue Decoction (QLD, *n* = 1), Qingri Jiedu Huoxue Decoction (QJHD, *n* = 1), Runzao Zhiyang Capsule (RZC, *n* = 3), Shufeng Jiedu Capsules (SJCs, *n* = 1), Total Glucosides of Paeony Capsules (TGPCs, *n* = 4), Tuiyin Decoction (TYD, *n* = 1), Xiaofeng Granules (XFGs, *n* = 1), Xiaofeng San (XFS, *n* = 1), Xiaoyin Decoction (XD, *n* = 2), Xiaoyin Granules1 (XG1, *n* = 1), Xiaoyin Granules2 (XG2, *n* = 1), Xiaoyin Granules3 (XG3, *n* = 1), Yinxie Capsule (YXC, *n* = 1), Zhuhuang Granules (ZGs, *n* = 1), Zidan Yinxie Granules (ZYDs, *n* = 2), and Ziyin Liangxue Decoction (ZLD, *n* = 1), respectively. 

In instances where it was apparent that the components differed despite the prescription name being identical, the researchers de facto applied their discretion to differentiate them by numbering each prescription name in the sequence 1, 2, 3, and so forth. The study duration ranged from 4 to 24 weeks, with 35 studies having a treatment duration of at least eight weeks. The interventions included pharmaceutical products (*n* = 16) and prescriptions for individualized studies (*n* = 24). The preparation type of EAHM by study was as follows: capsule (*n* = 10), decoction (*n* = 19), granules (*n* = 8), pills (*n* = 2), and powders (*n* = 1). A total of 31 studies reported on adverse events. The specific details of the individual drugs included in the EAHMs are provided in Supplementary Material S3A,B.

### 3.2. Risk of Bias Assessment

**Figure 2:**
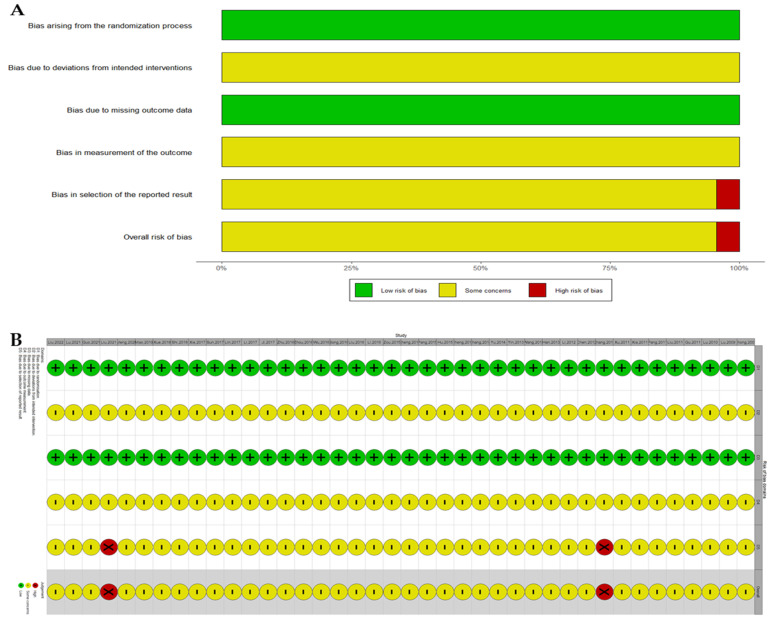
(**A**) Rob 2 summary plot; (**B**) Rob 2 traffic light plot.

The results of the risk of bias assessment of the 40 included studies are presented in Figure 2, which was generated using the R package robvis [82]. With regard to the criteria of “deviation from intended intervention” and “measurement of the outcome”, all studies were found to exhibit some degree of concern. The primary de facto reason for this assessment is that the double-blind design is challenging to implement in the context of combination therapy. Furthermore, none of the studies reflected elements such as investigator blinding in their design. Conversely, 38 studies were deemed to have some concern with regard to the selection of reported results. This was due to the fact that none of the studies had registered their study protocols in advance. Furthermore, two studies reported only one outcome and were classified as having a high risk of bias.

### 3.3. Pairwise Meta-Analysis

#### 3.3.1. Primary Outcomes

##### PASI 90

**Figure 3:**
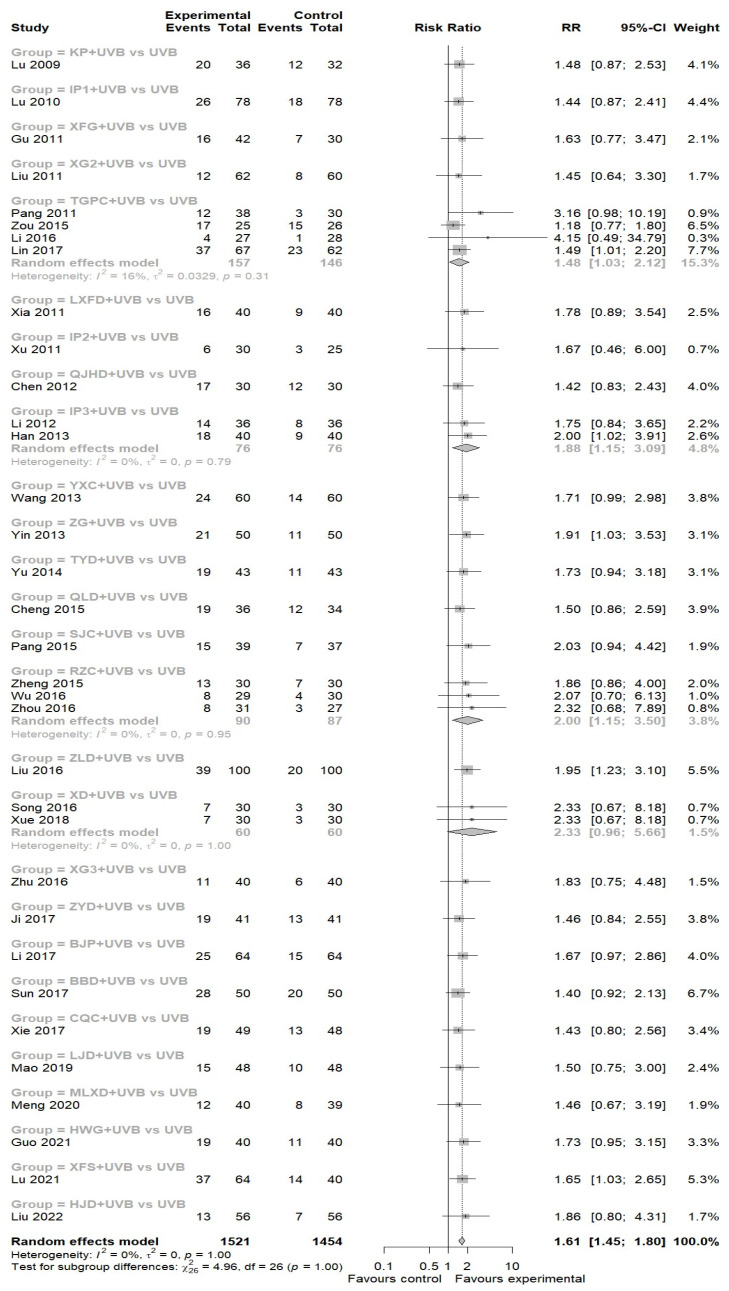
Forest plot for PASI90 in pairwise meta-analysis.

A total of 34 studies were conducted to assess the efficacy of EAHM in combination with NB-UVB versus NB-UVB monotherapy with PASI 90 [42,43,44,46,47,49,50,51,52,53,54,55,56,57,58,59,60,61,63,64,65,66,68,69,70,71,72,73,74,75,76,77,79,80]. The results demonstrated that the EAHM with NB-UVB arm exhibited a statistically significant improvement in PASI 90 achievement compared to the control arm (34 trials, *n* = 2975; RR: 1.6127; 95% CI: 1.4474 to 1.7967, *p* < 0.0001; heterogeneity *t*^2^ = 0, *I*^2^ = 0.0%, *p* = 1.0000; Figure 3). 

##### PASI 60

**Figure 4:**
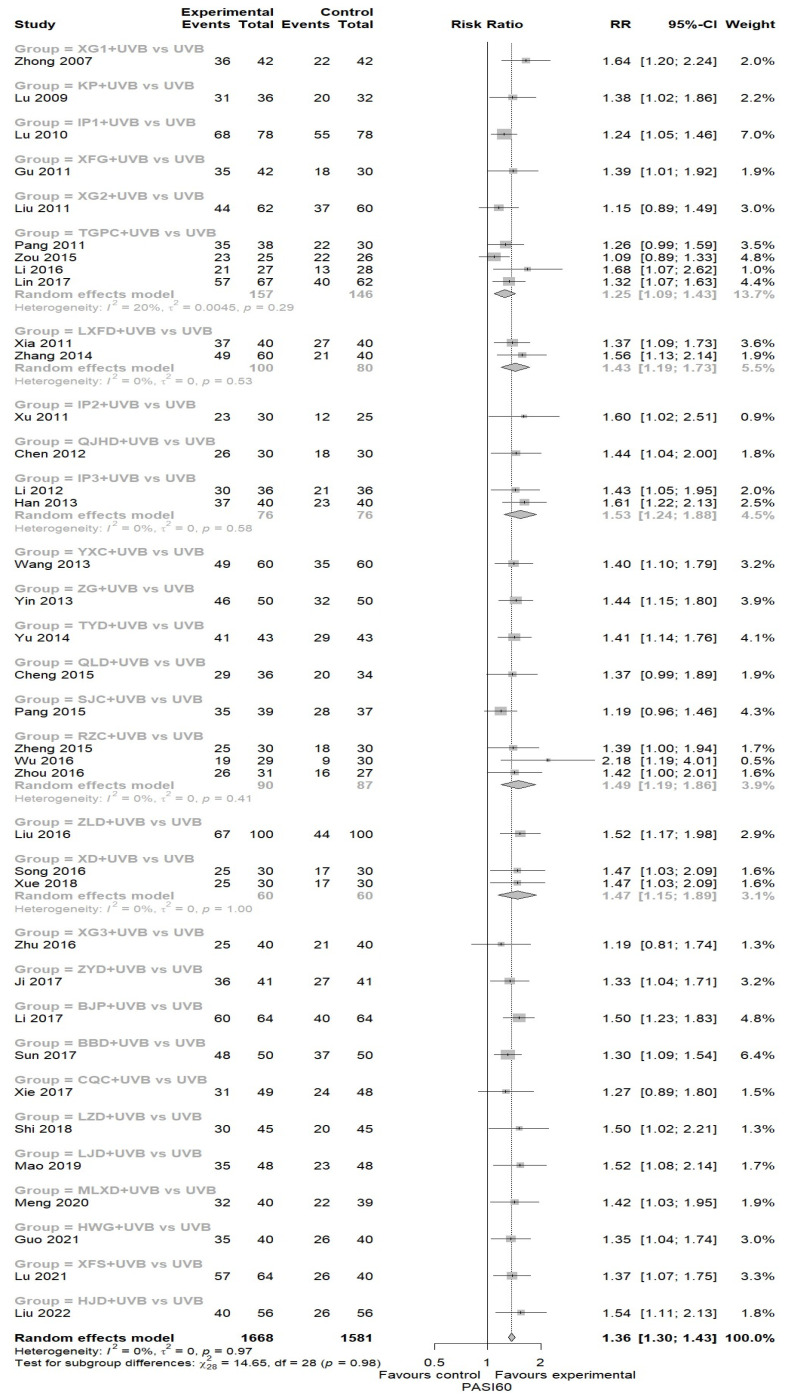
Forest plot for PASI60 in pairwise meta-analysis.

A total of 37 studies reported the effect of EAHM with NB-UVB combination therapy on PASI 60 [42,43,44,46,47,48,49,50,51,52,53,54,55,56,57,58,59,60,61,63,64,65,66,67,68,69,70,71,72,73,74,75,76,77,79,80,81]. The experimental arm demonstrated a statistically significant superiority compared to the NB-UVB monotherapy arm in achieving PASI 60 (37 trials, *n* = 3249; RR: 1.3637; 95% CI: 1.3050 to 1.4252, *p* < 0.0001; heterogeneity *t*^2^ = 0, *I*^2^ = 0.0%, *p* = 0.9696; Figure 4). 

##### PASI Score

**Figure 5:**
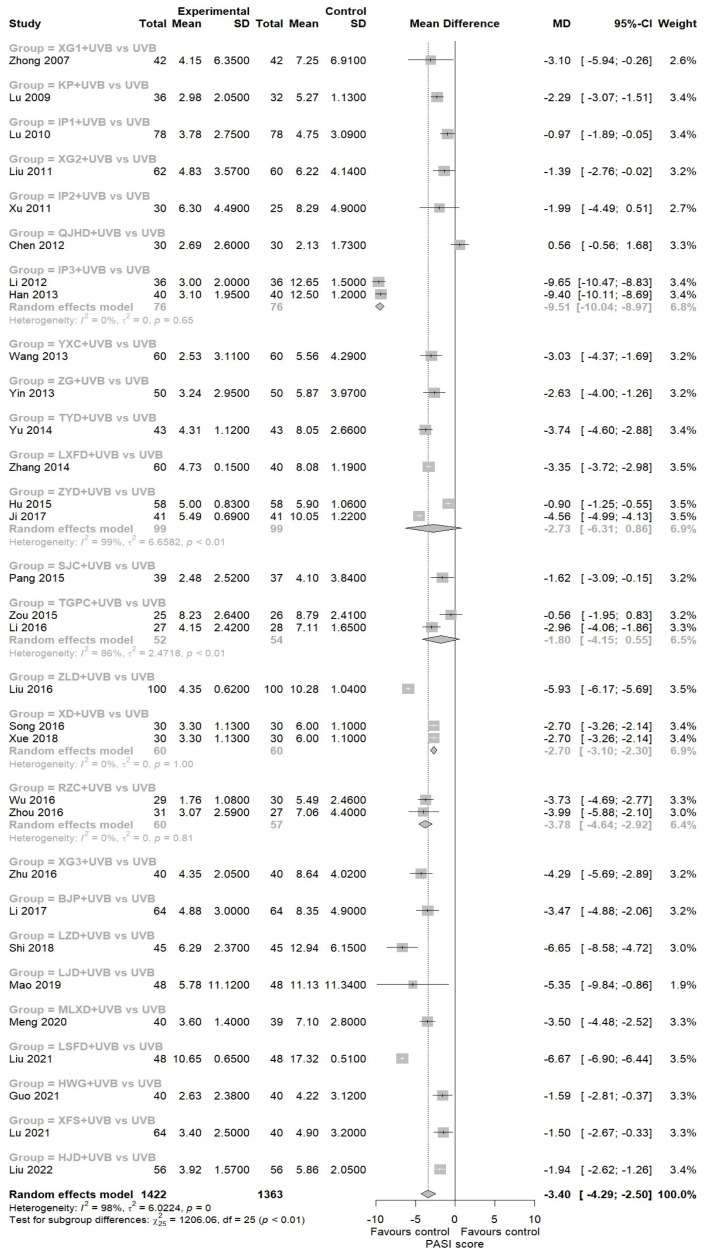
Forest plot for PASI score in pairwise meta-analysis.

A 31-study meta-analysis revealed that EAHM with NB-UVB combination therapy demonstrated significantly superior efficacy in reducing PASI scores compared to NB-UVB monotherapy [43,44,45,46,47,48,49,50,51,55,56,57,58,59,60,62,64,65,66,67,68,69,70,71,72,73,74,79,80,81]. However, the results also identified substantial heterogeneity (31 trials, *n* = 2785; MD: −3.3973; 95% CI: −4.2945 to −2.5001, *p* < 0.0001; heterogeneity *t*^2^ = 6.0224, *I*^2^ = 98.2%, *p* = 0; Figure 5). 

##### AEs Incidence Rate

**Figure 6:**
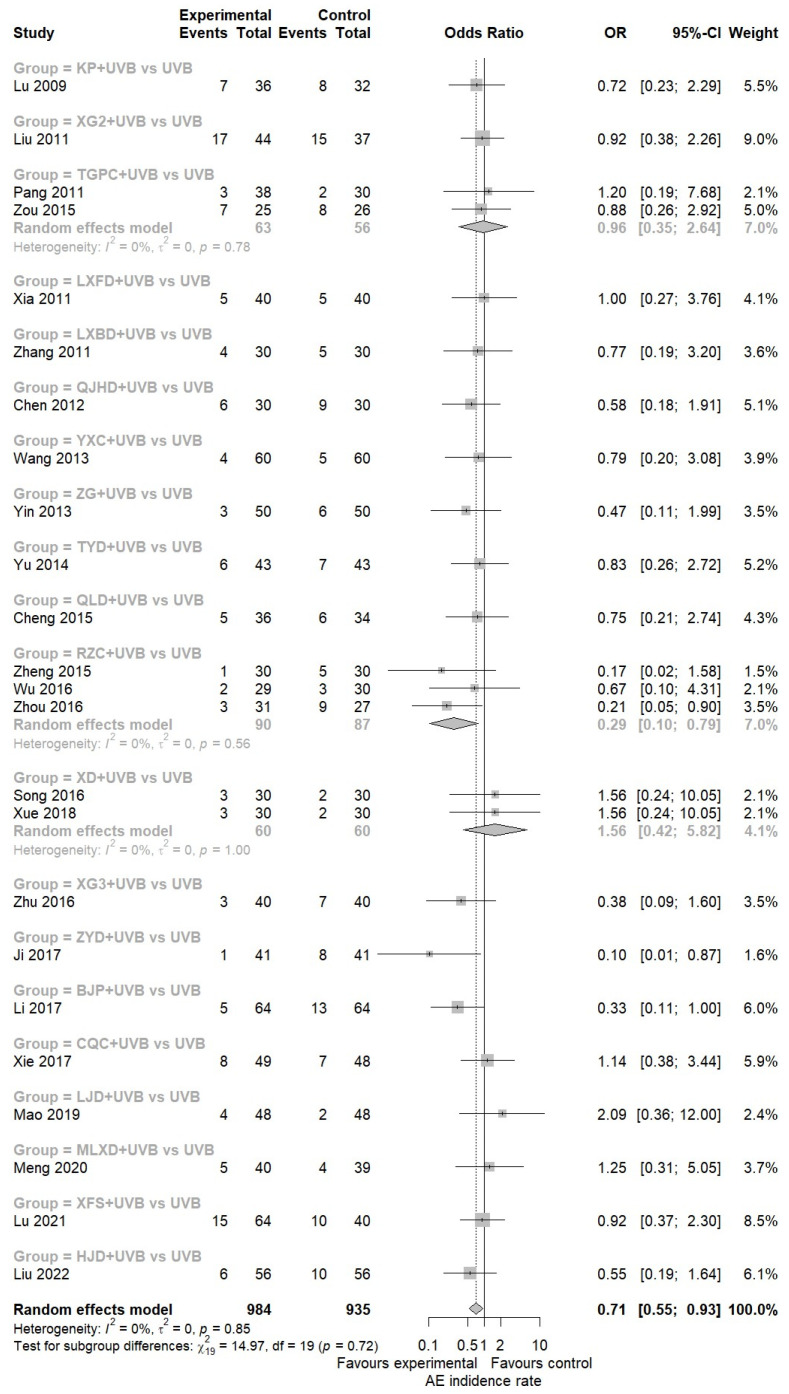
Forest plot for AEs incidence rate in pairwise meta-analysis.

In total, 24 studies were identified that reported AEs information for both the experimental and control groups analysis [42,43,46,47,49,50,51,54,56,57,58,59,61,63,65,66,68,70,71,73,76,77,78,80]. This allowed for the estimation of incidence rates. The results of the meta-analysis demonstrated that EAHM with NB-UVB combination therapy could significantly reduce the odds of AEs compared to NB-UVB monotherapy (24 trials, *n* = 1919; OR: 0.7136; 95% CI: 0.5455 to 0.9335, *p* < 0.0138; heterogeneity *t*^2^ = 0, *I*^2^ = 44.6%, *p* = 0.847; Figure 6). Meanwhile, the individual AEs documented in this study could be classified into one of two categories: gastrointestinal reactions or cutaneous symptoms, with the exception of one case of dizziness. Consequently, separate analyses were conducted on the incidence of AEs according to each category. The findings indicated that for gastrointestinal symptoms, the odds of AEs incidence were markedly higher in the EAHM combination group than in the NB-UVB group (15 trials; OR: 3.903; 95% CI: 2.4028 to 6.3426, *p* < 0.0001; heterogeneity *t*^2^ = 0, *I*^2^ = 0%, *p* = 0.9069; Supplementary Material S4A). In contrast, the favorable findings of the EAHM with NB-UVB combination therapy in the treatment of skin conditions were more pronounced than the overall AEs incidence comparison previously presented (24 trials; OR: 0.3401; 95% CI: 0.2312 to 0.5002, *p* < 0.0001; heterogeneity *t*^2^ = 0, *I*^2^ = 0%, *p* = 0.6559; Supplementary Material S4B). These findings are to be expected, given the disparate routes of administration and the distinctive characteristics of the EAHM and NB-UVB interventions.

#### 3.3.2. Secondary Outcomes

**Figure 7:**
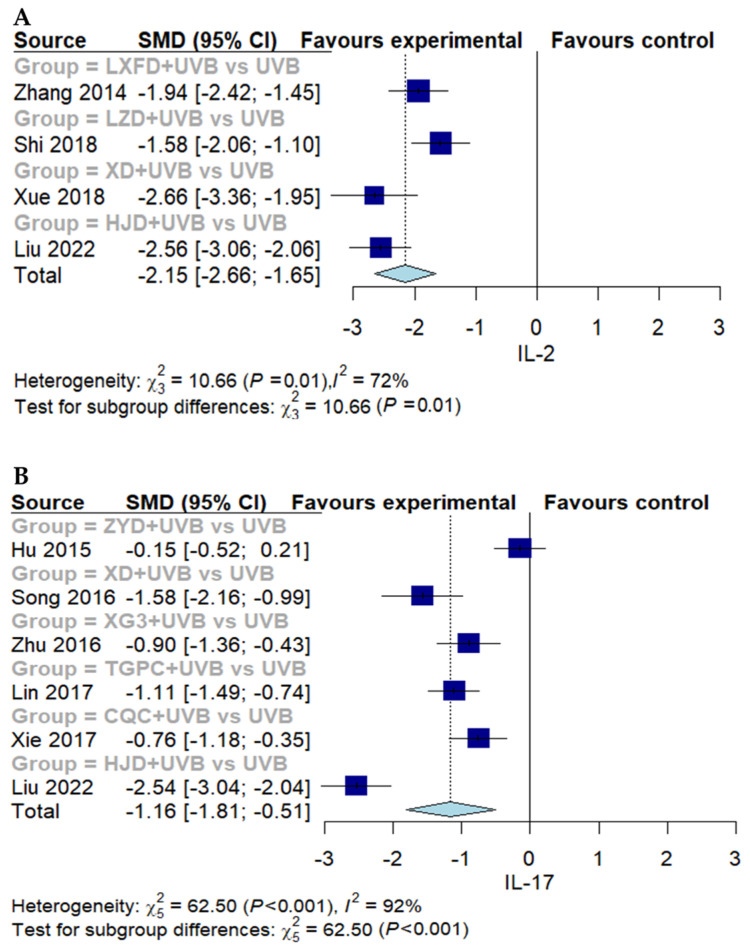

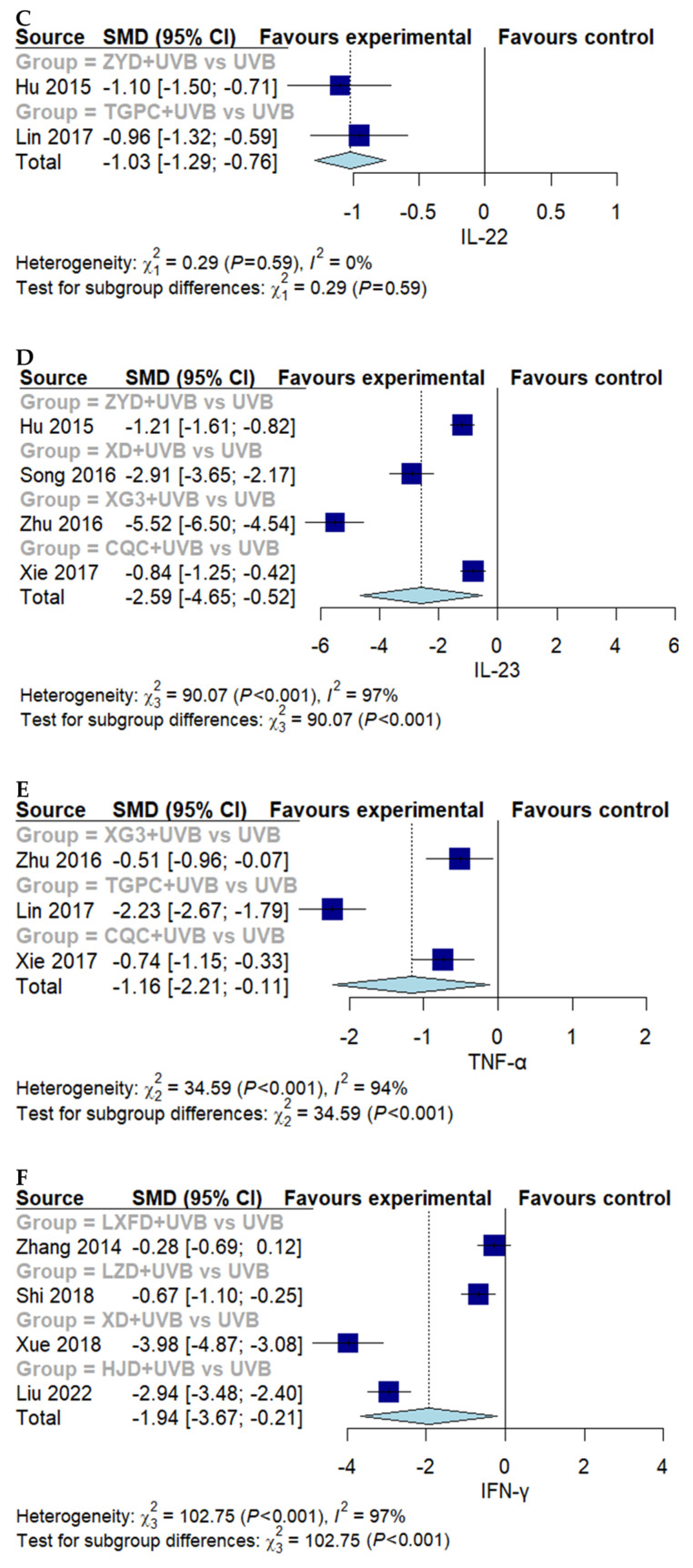

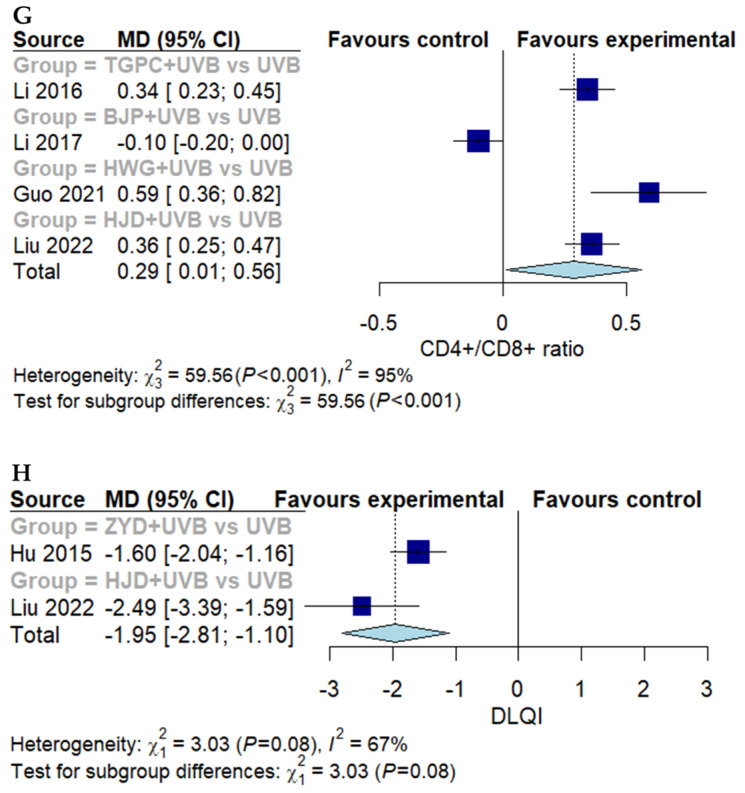
Forest plots for secondary outcomes in pairwise meta-analysis. (**A**) IL-2; (**B**) IL-17; (**C**) IL-22; (**D**) IL-23; (**E**) TNF-α; (**F**) IFN-γ; (**G**) CD4+/CD8+ratio; (**H**) DLQI.

The four studies that reported IL-2 measurements demonstrated that the combination of EAHM with NB-UVB was more effective in controlling immune-mediated inflammation (four trials, *n* = 362; SMD: −2.1540; 95% CI: −2.6651 to −1.6915, *p* < 0.0001; heterogeneity *t*^2^ = 0.1865, *I*^2^ = 71.9%, *p* = 0.0137; Figure 7A). The same trend was observed in the data related to IL-17 (six trials, *n* = 594; SMD: −1.1598; 95% CI: −1.8102 to −0.5093, *p* = 0.0005; heterogeneity *t*^2^ = 0.6072, *I*^2^ = 92.0%, *p* < 0.0001; Figure 7B), IL-22 (two trials, *n* = 245; SMD: −1.0260; 95% CI: −1.2903 to −0.7589, *p* < 0.0001; heterogeneity *t*^2^ = 0, *I*^2^ = 0%, *p* = 0.5899; Figure 7C), IL-23 (four trials, *n* = 353; SMD: −2.5860; 95% CI: −4.6503 to −0.5217, *p* = 0.0141; heterogeneity *t*^2^ = 4.3194, *I*^2^ = 96.7%, *p* < 0.0001; Figure 7D), TNF-α (three trials, *n* = 306; SMD: −1.1595; 95% CI: −2.2136 to −0.1055, *p* = 0.0311; heterogeneity *t*^2^ = 0.8187, *I*^2^ = 94.2%, *p* < 0.0001; Figure 7E), and IFN-γ (four trials, *n* = 362; SMD: −1.9396; 95% CI: −3.6686 to −0.2105, *p* = 0.0279; heterogeneity *t*^2^ = 3.0214, *I*^2^ = 97.1%, *p* < 0.0001; Figure 7F). In the meantime, four studies reported improvements in reversed CD4+/CD8+ ratios. A meta-analysis of these studies indicated that the EAHM with NB-UVB combination therapy may have a positive impact on improving the compromised immune system (four trials, *n* = 375; MD: 0.2875; 95% CI: 0.0118 to 0.5632, *p* = 0.0409; heterogeneity *t*^2^ = 0.0737, *I*^2^ = 95.0%, *p* < 0.0001; Figure 7G). Furthermore, the results of the DLQI, a widely utilized outcome measure with PASI, demonstrated partial support for the effect observed in the experimental arm for the primary outcome (two trials, *n* = 114; MD: −1.9548; 95% CI: −2.8089 to −1.1007, *p* < 0.0001; heterogeneity *t*^2^ = 0.2655, *I*^2^ = 67.0%, *p* = 0.2655; Figure 7H).

#### 3.3.3. Sensitivity Analysis

**Figure 8:**
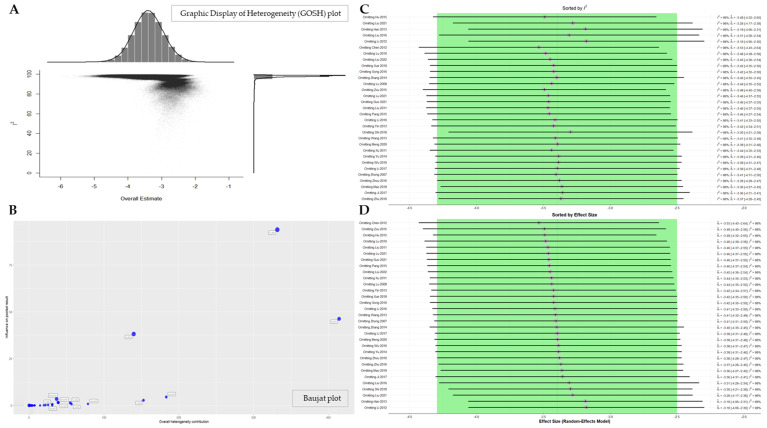
(**A**) GOSH plot; (**B**) Baujat plot; (**C**) forest plot of leave-one-out sensitivity analysis for PASI score, sorted by *I*^2^; (**D**) forest plot of leave-one-out sensitivity analysis for PASI score, sorted by effect size.

The meta-analysis results for PASI score exhibited considerable heterogeneity. To assess this heterogeneity, we initially performed a GOSH plot analysis (Figure 8A).

Unlike traditional sensitivity analysis, this method has the advantage of being able to fit all possible subsets and combinations of included studies. As illustrated in Figure 8A, the pooled effect sizes of the included studies are concentrated in the high-heterogeneity metrics. In order to identify individual studies that contribute disproportionately to the observed heterogeneity in the overall data, a Baujat plot was generated (Figure 8B). In the upper right corner of this diagnostic plot, we observe that the studies Liu 2011 [73], Hu 2015 [62], and Liu 2016 [55] have high values for both overall heterogeneity contribution and influence on the pooled results. However, upon exclusion of these key studies, a leave-one-out sensitivity analysis was conducted to re-examine their impact on the heterogeneity metric. The results of this analysis demonstrated that the exclusion of these three studies did not substantively alter the *I*^2^ value (Figure 8C,D). It can thus be concluded that the considerable heterogeneity observed in the PASI score is not attributable to the influence of a small number of studies but rather to the existence of differences in patient baseline conditions between the studies or differences in the composition of the individual EAHMs, which are essentially prescribed in accordance with a personalized dosing theory.

#### 3.3.4. Publication Bias

**Figure 9:**
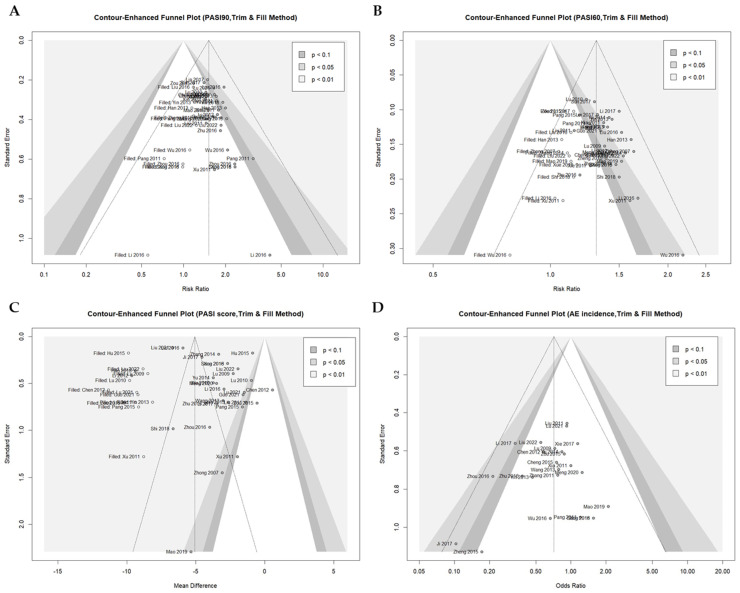
Contour-enhanced funnel plot with trim and fill method for (**A**) PASI 90; (**B**) PASI 60; (**C**) PASI score; (**D**) AEs incidence.

A contour-enhanced funnel plot and Egger’s test were employed to assess the potential for publication bias in the primary outcomes data (Figure 9A–D). A degree of asymmetry was visually apparent in the contour-enhanced funnel plots for PASI90 (t = 4.97, *df* = 32, *p* < 0.0001), PASI60 (t = 4.08, *df* = 35, *p* = 0.0003), and PASI score (t = 2.10, *df* = 29, *p*-value = 0.0442). Furthermore, Egger’s test demonstrated the existence of statistically significant publication bias for PASI90, PASI60, and PASI score, respectively. 

Nevertheless, no significant publication bias was observed in AEs incidence (t = −0.81, *df* = 22, *p*-value = 0.4259). In the three outcomes where publication bias was identified, the corrected effect estimate was recalculated by adjusting for the small-study effect using the trim and fill method. Even after correction of the estimates, PASI 90 (46 trials with 12 added studies, *n* = 3963; RR: 1.5140; 95% CI: 1.3724 to 1.6703, *p* < 0.0001), PASI 60 (49 trials with 12 added studies, *n* = 4363; MD: −5.0770; 95% CI: −5.9931 to −4.1609, *p* < 0.0001), and PASI score (43 trials with 12 added studies, *n* = 4363; RR: 1.3111; 95% CI: 1.2600 to 1.3642, *p* < 0.0001) all demonstrated a statistically significant comparative advantage of EAHM with NB-UVB combination therapy over NB-UVB monotherapy. Consequently, publication bias is evident in the primary outcomes data of this study; nevertheless, this is unlikely to negate the efficacy of the intervention.

#### 3.3.5. Quality of Direct Evidence

The quality of evidence for all outcomes for which direct evidence was generated through pairwise meta-analysis ranged from “low” to “moderate”. The details are summarized in Table 1 below.

### 3.4. Network Meta-Analysis 

#### 3.4.1. Characteristics of the Network

Given the considerable heterogeneity of the component herbs in individual EAHM prescriptions, we conducted a Bayesian network meta-analysis to form indirect evidence through comparisons between combination therapies. Table 2 provides a detailed summary of the information for each outcome, including the number of interventions and networks, the number of patients, whether networks were closed, and the number of direct comparisons.

#### 3.4.2. PASI90

**Figure 10:**
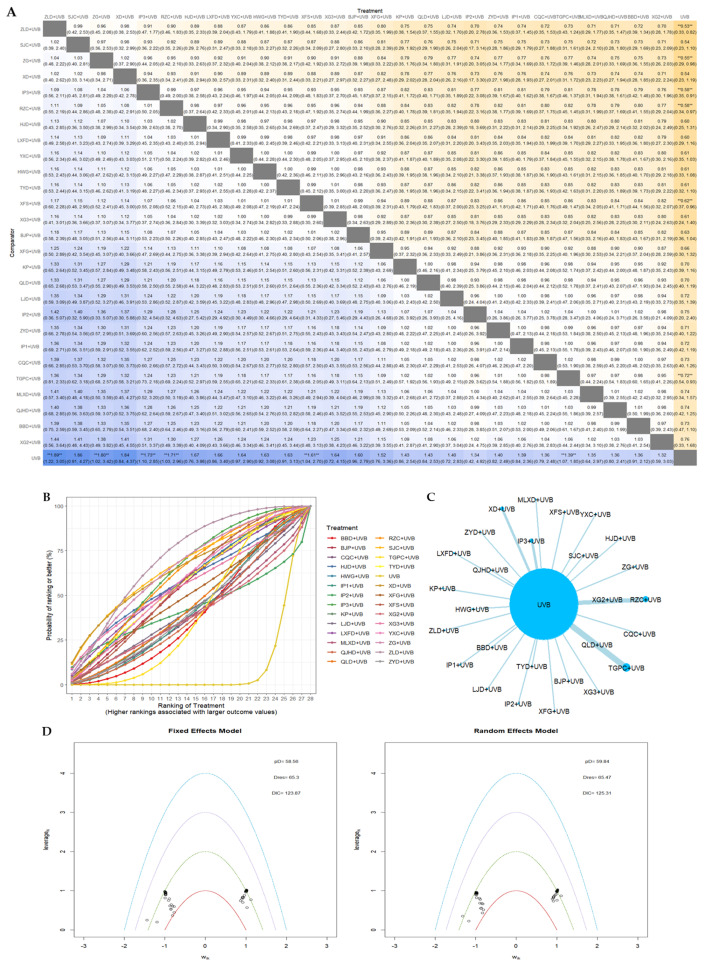
(**A**) League table heatmap for PASI90. The values displayed in each cell represent the relative treatment effect (and 95% confidence intervals) of the treatment on the top cell compared to the treatment on the left. The presence of a double asterisk indicates that the difference between the two treatments is statistically significant; (**B**) SUCRA plot for PASI 90; (**C**) network plot for PASI 90; (**D**) leverage plot and fit statistics for PASI 90. The model with the lower DIC value and fewer outliers in the leverage plot was selected by comparing the DIC values shown in the leverage plot.

The network relationships between interventions in the PASI90 network meta-analysis are shown in Figure 10C. In the comparison between models for PASI90, there was minimal visual distinction observed on the leverage plot. However, the DIC of the fixed-effects model (123.87) was found to be smaller than that of the random effects model (125.31), indicating that the former was selected for fitting and subsequent analysis (Figure 10D). The SUCRA values and ranks for each EAHM and NB-UVB combination were as follows: ZLD + UVB (rank 1, 70.8%) > SJCs + UVB (rank 2, 66.18%) > ZGs + UVB (rank 3, 65.16%) > XD + UVB (rank 4, 64.1%) > IP3 + UVC (rank 5, 63.19%) > RZC + UVB (rank 6, 61.49%) > HJD + UVB (rank 7, 57.84%) > LXFD + UVB (rank 8, 57.55%) > YXC + UVB (rank 9, 57.35%) > HWG + UVB (rank 10, 57.09%) > TYD + UVB (rank 11, 56.61%) > XFS + UVB (rank 12, 55.89%) > XG3 + UVB (rank 13, 55.5%) > BJPs + UVB (rank 14, 55.02%) > XFGs + UVB (rank 15, 50.2%) > QLD + UVB (rank 16, 44,63%) > KP + UVB (rank 17, 43.95%) > LJD + UVB (rank 18, 42.86%) > ZYDs + UVB (rank 19, 42.58%) > IP2 + UVB (rank 20, 42.44%) > IP1 + UVB (rank 21, 41.71) > MLXD + UVB (rank 22, 40.78%) > TGPCs + UVB (rank 23, 40.37%) > CQC + UVB (rank 24, 40.06%) > QJHD + UVB (rank 25, 39.44%) > XG2 + UVB (rank 26, 39.26%) > BBD + UVB (rank 27, 38.99%) > UVB (rank 28, 8.97%). The League heat plot and SUCRA plot for these results are presented as Figure 10A and Figure 10B, respectively. However, statistically significant differences were found between the following comparisons: ZLD + UVB vs. UVB (RR 1.89; 95% CrI 1.22 to 3.05), ZGs + UVB vs. UVB (RR 1.80, 95% CrI 1.02 to 3.42), IP3 + UVC vs. UVB (RR 1.73, 95% CrI 1.10 to 2.85), RZC + UVB vs. UVB (RR 1.71, 95% CrI 1.03 to 2.96), XFS + UVB vs. UVB (RR 1.61, 95%CrI 1.04 to 2.70), TGPCs + UVB vs. UVB (RR 1.39, 95% CrI 1.07 to 1.85). In PASI 90, the interventions that demonstrated statistical significance and were ranked in the top 10 on SUCRA were ZLD + UVB, ZGs + UVB, IP3 + UVC, and RZC + UVB.

#### 3.4.3. PASI60

**Figure 11:**
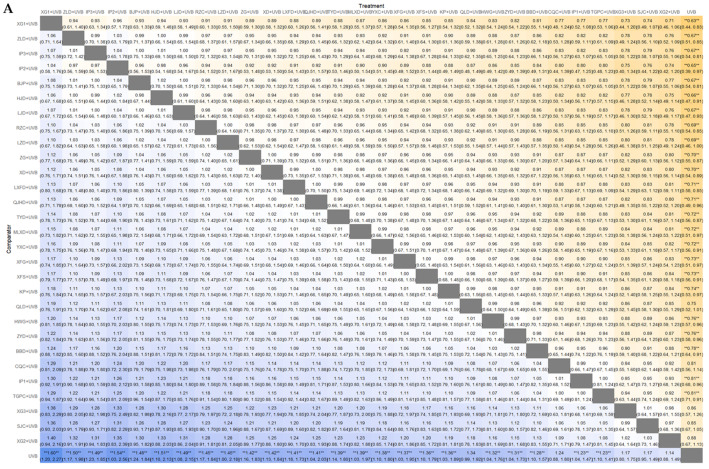

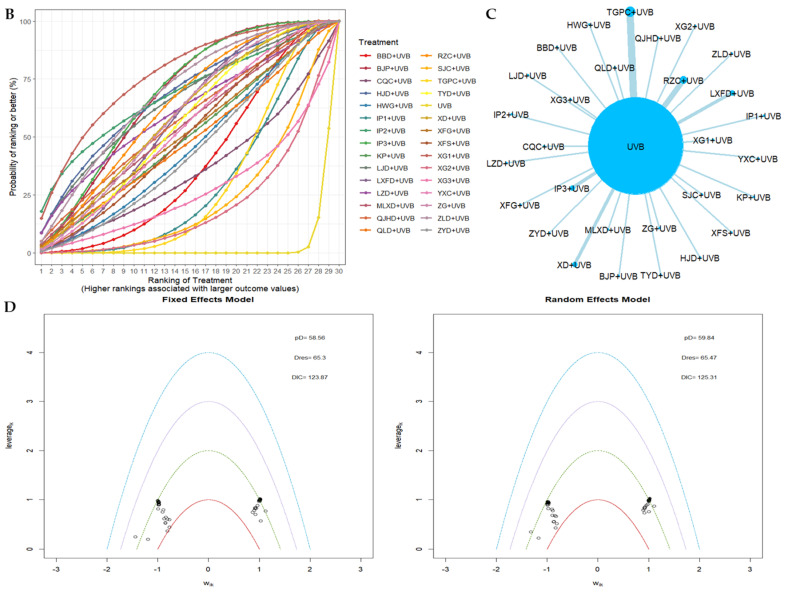
(**A**) League table heatmap for PASI60. The values displayed in each cell represent the relative treatment effect (and 95% confidence intervals) of the treatment on the top cell compared to the treatment on the left. The presence of a double asterisk indicates that the difference between the two treatments is statistically significant; (**B**) SUCRA plot for PASI60; (**C**) network plot for PASI60; (**D**) leverage plot and fit statistics for PASI60. The model with the lower DIC value and fewer outliers in the leverage plot was selected by comparing the DIC values shown in the leverage plot.

The network geometry between interventions in the network meta-analysis of PASI60 outcome is depicted in Figure 11C. In the model comparison conducted in PASI 60, the fixed-effects model exhibited a smaller DIC value than the random effects model. Consequently, the former was selected as the optimal model (Figure 11D). The SUCRA values and ranks for each EAHM and NB-UVB combination were as follows: XG1 + UVB (rank 1, 77.03%) > ZLD + UVB (rank 2, 68.91%) > IP3 + UVB (rank3, 68.78%) > IP2 + UVB (rank 4, 68.37%) > BJPs + UVB (rank 5, 68.18%) > HJD + UVB (rank 6, 67.88%) > LJD + UVB (rank 7, 65.5%) > RZC + UVB (rank 8, 63.38%) > LZD + UVB (rank 9, 60.88%) > ZGs + UVB (rank 10, 59.19%) > XD + UVB (rank 11, 59.08%) > LXFD + UVB (rank 12, 57.79%) > QJHD + UVB (rank 13, 57.28%) > TYD + UVB (rank 14, 56.02%) > MLXD + UVB (rank 15, 54.68%) > YXC + UVB (rank 16, 53.53%) > XFGs + UVB (rank 17, 51.58%) > XFS + UVB (rank 18, 51.58%) > KP + UVB (rank 19, 49.88%) > QLD + UVB (rank 20, 48.02%) > HWG + UVB (rank 21, 45.45) > ZYDs + UVB (rank 22, 43.99%) > BBD + UVB (rank 23, 38.56%) > CQC + UVB (rank 24, 35.33%) > IP1 + UVB (rank 25, 29.12%) > TGPCs + UVB (rank 26, 28.76%) > XG3 + UVB (rank 27, 26.92%) > SJCs + UVB (rank 28, 22.95%) > XG2 + UVB (rank 29, 19.65%) > UVB (rank 30, 2.49). The League heat plot and SUCRA plot for these results are presented as Figure 11A and Figure 11B, respectively. While the majority of interventions demonstrated statistical significance in comparison with UVB, no statistical significance was observed in the comparison between EAHM combination therapies. In PASI 60, the interventions that demonstrated statistical significance and were ranked in the top 10 on SUCRA were XG1 + UVB, ZLD + UVB, IP3 + UVB, IP2 + UVB, BJPs + UVB, HJD + UVB, LJD + UVB, RZC + UVB, LZD + UVB, and ZGs + UVB. 

#### 3.4.4. PASI Score

**Figure 12:**
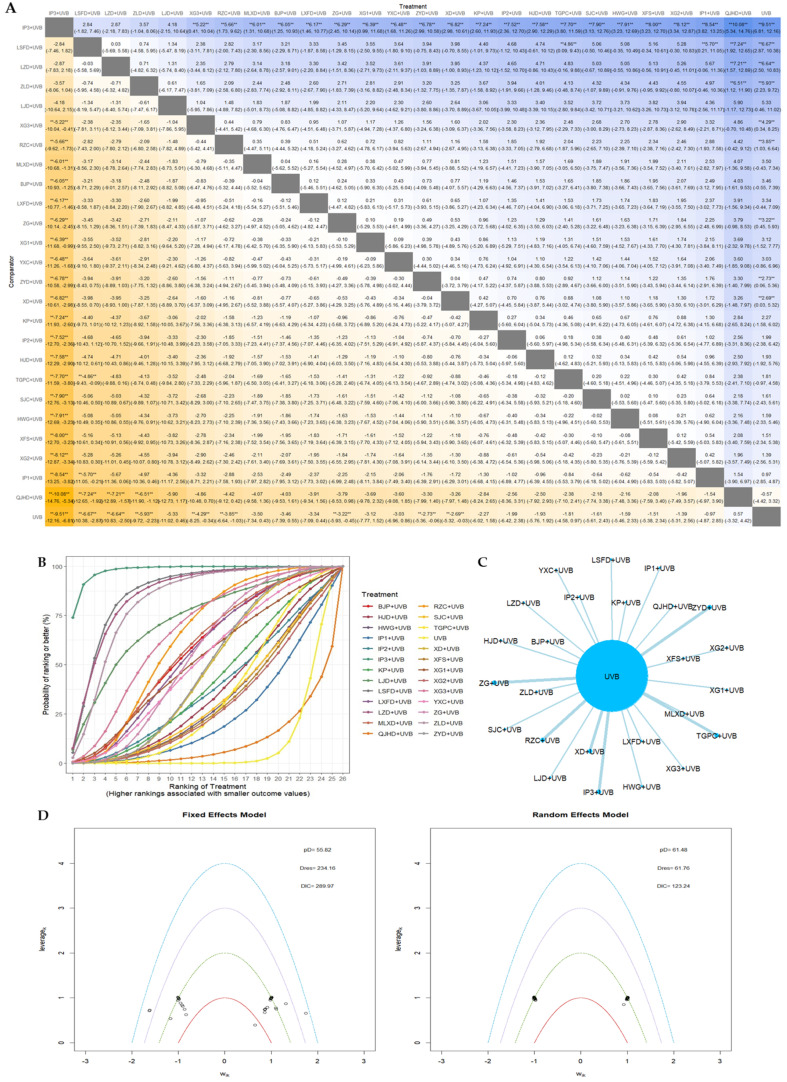
(**A**) League table heatmap for PASI score. The values displayed in each cell represent the relative treatment effect (and 95% confidence intervals) of the treatment on the top cell compared to the treatment on the left. The presence of a double asterisk indicates that the difference between the two treatments is statistically significant; (**B**) SUCRA plot for PASI score; (**C**) network plot for PASI score; (**D**) leverage plot and fit statistics for PASI score. The model with the lower DIC value and fewer outliers in the leverage plot was selected by comparing the DIC values shown in the leverage plot.

The network geometry between interventions in the network meta-analysis of PASI score outcome is depicted in Figure 12C. In the model fit comparison, the DIC of the fixed-effects model (289.97) is considerably larger than that of the random effects model (123.24). Therefore, the latter was selected as the model for the PASI score analysis (Figure 12D). The SUCRA values and ranks for each EAHM and NB-UVB combination were as follows: IP3 + UVB (rank 1, 98.14%) > LSFD + UVB (rank 2, 86.97%) > LZD + UVB (rank3, 85.99%) > ZLD + UVB (rank 4, 82.44%) > LJD + UVB (rank 5, 72.63%) > XG3 + UVB (rank 6, 66.82%) > RZC + UVB (rank 7, 63.31%) > MLXD + UVB (rank 8, 57.75%) > BJPs + UVB (rank 9, 56.96%) > LXFD + UVB (rank 10, 55.7%) > ZGs + UVB (rank 11, 54.88%) > XG1 + UVB (rank 12, 51.93%) > YXC + UVB (rank 13, 51.34%) > ZYDs + UVB (rank 14, 47.58%) > XD + UVB (rank 15, 46.96%) > KP + UVB (rank 16, 41.09%) > IP2 + UVB (rank 17, 37.95%) > HJD + UVB (rank 18, 36.53%) > TGPCs + UVB (rank 19, 33.99%) > SJCs + UVB (rank 20, 32.65%) > HWG + UVB (rank 21, 32.3) > XFS + UVB (rank 22, 31.1%) > XG2 + UVB (rank 23, 29.78%) > IP1 + UVB (rank 24, 24.51%) > QJHD + UVB (rank 25, 10.61%) > UVB (rank 26, 10.05%). The League heat plot and SUCRA plot for these results are presented as Figure 12A and Figure 12B, respectively. Among the interventions ranked within the top 10, IP3 + UVB, LSFD + UVB, LZD + UVB, ZLD + UVB, XG3 + UVB, and RZC + UVB demonstrated a statistically significant improvement in PASI score. It is noteworthy that IP3 + UVB exhibited a comparative advantage in PASI score effectiveness over all other interventions, with the exception of LSFD + UVB, LZD + UVB, ZLD + UVB, and LJD + UVB.

#### 3.4.5. AEs Incidence Rate

**Figure 13:**
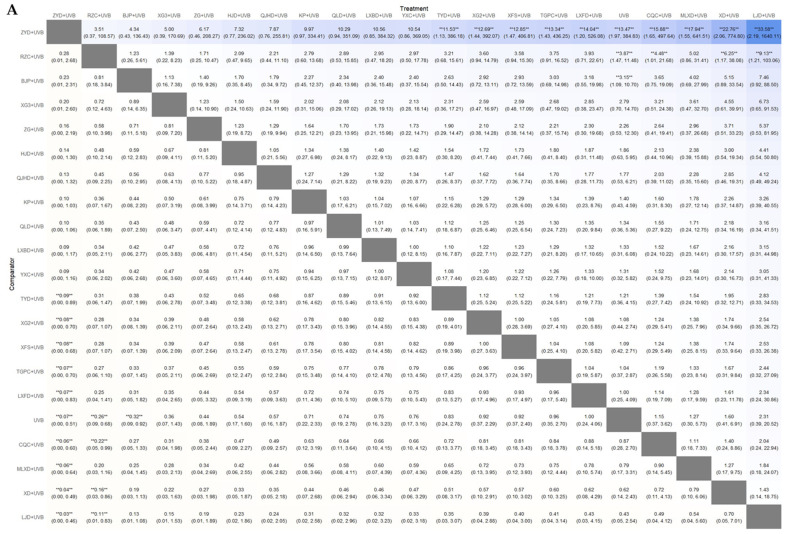

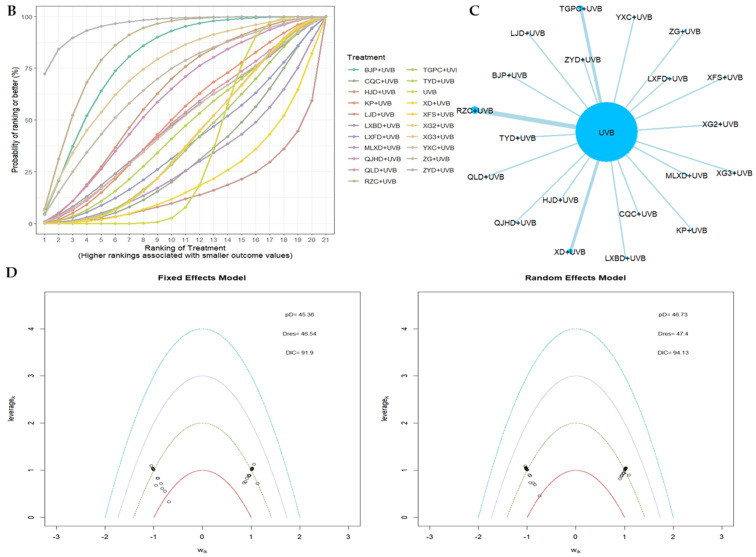
(**A**) League table heatmap for AEs incidence. The values displayed in each cell represent the relative treatment effect (and 95% confidence intervals) of the treatment on the top cell compared to the treatment on the left. The presence of a double asterisk indicates that the difference between the two treatments is statistically significant; (**B**) SUCRA plot for AEs incidence; (**C**) network plot for AEs incidence; (**D**) leverage plot and fit statistics for AEs incidence. The model with the lower DIC value and fewer outliers in the leverage plot was selected by comparing the DIC values shown in the leverage plot.

The network geometry between interventions in the network meta-analysis of AEs incidence is presented in Figure 13C. In the model comparison for the analysis of the AEs incidence outcome, the DIC of the fixed-effects model (91.9) was found to be lower than that of the random effects model. Consequently, the former was selected for further analysis (Figure 13D). The SUCRA values and ranks for each EAHM and NB-UVB combination were as follows: ZYDs + UVB (rank 1, 96.15%) > RZC + UVB (rank 2, 85.04%) > BJPs + UVB (rank3, 79.44%) > XG3 + UVB (rank 4, 73.67%) > ZGs + UVB (rank 5, 67.31%) > HJD + UVB (rank 6, 61.97%) > QJHD + UVB (rank 7, 59.57%) > KP + UVB (rank 8, 50.31%) > QLD + UVB (rank 9, 48.89%) > LXBD + UVB (rank 10, 48.31%) > YXC + UVB (rank 11, 47.99%) > TYD + UVB (rank 12, 44.64%) > XG2 + UVB (rank 13, 39.54%) > XFS + UVB (rank 14, 39.48%) > TGPCs + UVB (rank 15, 38.02%) > LXFD + UVB (rank 16, 37.59%) > UVB (rank 17, 34.32%) > CQC + UVB (rank 18, 31.32%) > MLXD + UVB (rank 19, 29.24%) > XD + UVB (rank 20, 21.42%) > LJD + UVB (rank 21, 15.67). The League heat plot and SUCRA plot for these results are presented as Figure 13A and Figure 13B, respectively. Of the interventions ranked in the top 10, three were statistically significantly superior in terms of reducing the AEs incidence: ZYDs + UVB, RZC + UVB, and BJPs + UVB. In contrast, four interventions ranked lower than UVB monotherapy: CQC + UVB, MLXD + UVB, XD + UVB, and LJD + UVB, which did not statistically significantly increase the probability of AEs compared to UVB monotherapy.

#### 3.4.6. Inconsistency Test

**Figure 14:**
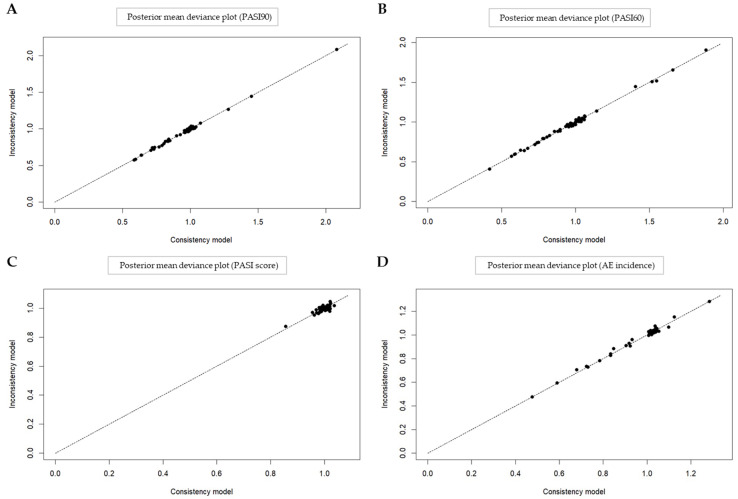
Posterior mean deviance plot for inconsistency test (**A**) PASI90; (**B**) PASI60; (**C**) PASI score; (**D**) AEs incidence. The data points represent the contribution of each treatment arm to the posterior mean deviance for both the consistency and inconsistency models.

In order to ascertain the existence of inconsistencies within the dataset analyzed by Bayesian network meta-analysis, a separate model was fitted assuming the presence of inconsistencies. The posterior mean deviance contributions of individual data points were plotted against the original model (Figure 14A–D). The figures demonstrate that there is no evidence of inconsistency within the network, indicating that the consistency assumption is not violated for all outcomes.

### 3.5. Network Analysis of the Individual Drugs Comprising an EAHM Prescription

**Figure 15:**
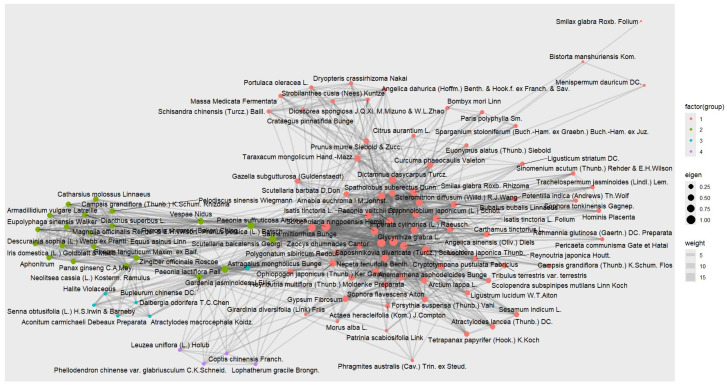
Network relationships of all herbs used as component drugs in EAHM and NB-UVB combination therapy. Each color-coded shade represents herbs that are close in the network topology.

To identify herbs in EAHM formulations that are likely to be important contributors to psoriasis treatment efficacy based on PageRank centrality, network analysis was performed on all individual herbs used as interventions in the studies included in this review (Figure 15). The analysis showed that the total number of herbs used in clinical trials was 105, and the calculated PageRank centrality ranged from 0.0018 to 0.0256. The top 10 most key component herbs based on centrality were *Paeonia suffruticosa* Andrews (0.0256), *Smilax glabra* Roxb. Rhizoma (0.0235), *Salvia miltiorrhiza* Bunge (0.0232), *Glycyrrhiza glabra* L. (0.0229), *Rehmannia glutinosa* (Gaertn.) DC. Recens (0.0228), *Scutellaria baicalensis* Georgi (0.0197), *Paeonia lactiflora* Pall. (0.0188), *Lonicera japonica* Thunb. (0.0181), *Dictamnus dasycarpus* Turcz. (0.0177), and *Arnebia euchroma* I.M.Johnst. (0.0177), respectively. The full list of individual drugs analyzed, their centrality rankings, and the PageRank centrality value computed for each drug can be found in Supplementary Material S5.

## 4. Discussion

### 4.1. Summary of Findings

The key findings from our analyses are summarized as follows: Pairwise meta-analysis demonstrated that the combination therapy of EAHM with NB-UVB was superior to NB-UVB monotherapy in terms of efficacy and safety. This was supported by primary outcomes including the PASI and the AEs incidence. Further analysis showed that EAHM significantly reduced the likelihood of cutaneous AEs during NB-UVB treatment. Secondary outcomes, including several hematological and immune-mediated inflammatory markers and Dermatology Life Quality Index (DLQI) scores, corroborated these favorable results. Bayesian meta-analysis further demonstrated the benefits of EAHM with NB-UVB combination therapy over NB-UVB monotherapy, aligning with the frequentist-based pairwise meta-analysis. The relative superiority of each EAHM formulation based on SUCRA values was also derived, but the statistical differences between them were not significant. However, ZLD with NB-UVB and IP3 with NB-UVB were consistently in the top five for PASI90, PASI60, and PASI score improvements. In terms of AEs incidence, ZYDs with NB-UVB showed a statistically meaningful safety advantage over many other interventions. The most common dosage form of EAHM analyzed in this study was decoction, but other dosage forms such as granules, pills, and capsules were also used. Future studies could analyze the superiority of different dosage forms, but more evidence is needed. The next highest intervention, RZC with NB-UVB, had overall SUCRA scores in the top 10 for safety and efficacy. Network analysis of 105 herbs in the EAHM formulae provided a predictive ranking of the influence of individual herbs by PageRank centrality. The top five herbs include *Paeonia suffruticosa* Andrews, *Smilax glabra* Roxb. Rhizoma, *Salvia miltiorrhiza* Bunge, *Glycyrrhiza glabra* L., *Rehmannia glutinosa* (Gaertn.) DC. Recens. These findings have implications for the treatment of psoriasis and warrant further discussion.

### 4.2. Strength and Weakness

This study represents a step forward from previous research conducted in 2015 on the efficacy of EAHM combined with NB-UVB therapy for the treatment of psoriasis [27]. To the best of our knowledge, this is the first Bayesian network meta-analysis of the efficacy and safety of oral EAHM with NB-UVB combination therapy. The number of analyzable RCTs has more than doubled from 18 to 40, while the sample size of the participants has increased significantly from 1342 to 3521. This increase in data has allowed for more detailed analyses and more robust conclusions, underpinned by increased statistical power. Our adoption of Bayesian network meta-analysis was driven by the need to take full advantage of this expanded dataset. Bayesian statistical methods are particularly advantageous in this context because they are well suited to generating estimates from relatively small datasets and allow inferences to be continuously updated as new data become available. This approach has allowed us to build on and further strengthen the findings of the 2015 study, providing more evidence that EAHM combined with NB-UVB therapy provides superior benefits to psoriasis patients compared to NB-UVB monotherapy alone. A key contribution of our study is the comparative effectiveness analysis of different EAHM prescriptions. This analysis has revealed insights into specific EAHM formulations with comparative advantages, which may inform future research designs and clinical decision-making in psoriasis treatment. In addition, we implemented an exploratory approach to analyzing the complex nature of EAHM by conducting a network topology analysis of the component herbs. In contrast to more straightforward approaches such as frequency analysis or other centrality indices, the PageRank centrality methodology, which was employed in this study, enables the simultaneous assessment of the frequency of combinations of different herbs in the treatment of psoriasis and the extent to which the herbs act as key connectors within the formulation. The herbs that ranked highly in our centrality-based analysis can be inferred to play particularly crucial roles in the overall therapeutic effect of these complex herbal formulations. This insight opens up new avenues for understanding the mechanisms of action in EAHM and may guide future research into optimizing herbal combinations for psoriasis treatment. It is noteworthy that ZLD and IP3, which are anticipated to exhibit a potential superiority in terms of efficacy, have in common a number of key drugs that are regarded as being of relative importance, including *Rehmannia glutinosa* (Gaertn.) DC. Recens, *Salvia miltiorrhiza* Bunge, *Salvia miltiorrhiza* Bunge, and *Glycyrrhiza glabra* L. This observation appears to provide partial support for the aforementioned assumption.

Despite these positive features, the results of this study should be interpreted with caution, as a robust evidence base has not yet been established. First, additional studies are needed to confirm statistically significant differences in efficacy between EAHMs, as suggested by the Bayesian network meta-analysis. The current analysis, which includes only one trial per EAHM, does not allow firm conclusions about relative efficacy to be drawn. The analysis shows a substantial underpowering of the trials, especially with regard to safety improvements with combination therapy. Even if additional analyses show meaningful results in this study, it is important to note that these are subgroup analyses and therefore observational rather than randomized experimental results. Second, there is an urgent need to improve the quality of current RCTs, in parallel with increasing the quantity of the data. The substantial increase in available clinical data compared with a decade ago has not led to clearer conclusions because the quality of individual RCT designs has scarcely improved. No study was rated as having an overall low risk of bias in the RoB assessment, and the GRADE-based certainty of the direct evidence was mostly rated as low. This reflects the current state of research in this area. Therefore, randomized trials with double-blind, placebo-controlled, multicenter designs are essential for more reliable decision-making in the future. Furthermore, given the dual nature of this study as both a clinical trial and a systematic review, further statistical testing of the EAHM prescriptions and the estimated relative benefits of individual herbs presented here should be conducted in separate studies. Future research with designs that include additional validation of findings, such as in vitro or in vivo studies where feasible, would provide a more comprehensive evidence base that better addresses the characteristics of EAHMs.

### 4.3. Implications of Clinical Practice

Despite the several limitations discussed above, this study can inform clinical decision-making in several ways. First, the results are based on objectively reliable and valid outcomes rather than total effectiveness rates, which are often controversial in existing EAHM studies. A large body of literature supports the efficacy and safety of EAHM as an adjunct to conventional therapy for psoriasis [21,23,24]. In particular, a separate systematic review published in 2017 highlighted the value of combining EAHM with NB-UVB as a topical, non-oral treatment [83]. The findings of the present study are consistent with these previous studies in terms of results and direction, suggesting that despite its challenges, EAHM is worth considering as an adjunct to various conventional therapies, including NB-UVB, in the treatment of psoriasis. Most studies of EAHM combination therapy to date have measured its efficacy and safety as a topical agent, such as in bath therapy. With this in mind, the current study may help to inform decisions regarding the use of EAHM as a combination with conventional treatment for psoriasis in regions where it is available as an orally administered drug. Notably, NB-UVB is often associated with cutaneous AEs such as erythema, pruritus, burning, xerosis, stinging and blistering, which may reduce treatment adherence. Short-term combination therapy with EAHM may improve this situation [10]. In addition, frequent clinic visits are not cost-effective for patients who are not responsive to NB-UVB alone. EAHMs may address this issue in cases where there are cost or safety barriers to systemic agents [11].

### 4.4. Implications of Preclinical Research

As mentioned above, EAHMs are prescribed in combination form according to a theory that seeks to maximize synergy through the multi-compound, multi-target action of different herbs [14,84]. This theory is referred to as “Gun-Shin-Jwa-Sa” (sovereign–minister–assistant–courier in standard WHO terminology), which refers to the principle of optimizing efficacy based on the distribution of roles among the herbs being combined. In the case of ZLD and IP3, which consistently showed the highest trend in efficacy in this study’s network meta-analysis, the constituent drugs included four and five of the top five drugs in terms of network centrality, respectively. In addition, *Rehmannia glutinosa* (Gaertn.) DC Recens and *Paeonia × suffruticosa* Andrews are herbs that have been reported to be actively used in combination therapy with orally administered conventional medicines [21]. Both in the context of long-term use and recent clinical data, EAHMs are considered to be more effective based on the interrelationships of drug–drug networks that form specific indications, rather than as individual herbs [85]. Thus, the herbs with the highest PageRank centrality in this study are believed to play a role in this regard, supporting the observed efficacy and safety of the results.

The herbs ranked in the top five for centrality are those that have the potential to effectively inhibit immune-mediated inflammation in psoriasis. The top-ranked herb, *Paeonia × suffruticosa* Andrews, has been shown in several studies to have potent anti-inflammatory effects, and recent research is unraveling its mechanism of action by simultaneously inhibiting the release of inflammatory factors, the expression of inflammatory genes, and the activity of enzymes involved in inflammation [86]. A recent study identified this herb as a nuclear factor-κB inhibitor and reported its anti-inflammatory effects as a multi-component activity, including paeoniflorin, oxypaeoniflorin, galloyl paeoniflorin, benzo-yloxypaeoniflorin, mudanpioside C, paeonol, and gallic acid [87]. Of these, paeonol, a major constituent of *Paeonia × suffruticosa* Andrews, has been shown to inhibit skin inflammation and pruritus in an experimental dry skin mouse model [88]. 

*Smilax glabra* Roxb. Rhizoma also has a long history of use in inflammatory skin conditions based on the action of several active constituents, including astilbin, isoengelitin, neoisoastilbin, neoastilbin, astragaloside, diosgenin, resveratrol, stigmasterol, β-sitosterol, and quercetin [89]. For example, the total glucosides of *Smilax glabra* Roxb. Rhizoma may have an effect on psoriasis by downregulating the Th17/Treg ratio and reducing the proportion of G2 phase cells in skin lesions in an imiquimod (IMQ)-induced psoriasis model [90]. The above previous study also found that total glucosides from *Smilax glabra* Roxb. Rhizoma significantly improved symptoms such as tail scale, skin erythema, and ulceration in a psoriasis-induced in vivo model and concluded that psoriasis could be a new indication for this herb. Meanwhile, a metabolomics and RNA-seq study published in 2022 also reported that *Smilax glabra* Roxb. Rhizoma could effectively alleviate dermatitis in an IMQ-induced psoriasis model when combined with *Actaea heracleifolia* (Kom.) J. Compton [91]. In addition, the study explained that the above effects were mediated by a multi-targeted mechanism of action, including the inhibition of cytokine and chemokine expression in the MAPK pathway and improvement in amino acid and carnitine metabolism in an in vivo model. The flavonoid astilbin is a major active constituent of *Smilax glabra* Roxb. Rhizoma was also shown to inhibit pathophysiology, including IMQ-induced keratinocyte proliferation, infiltration of CD3+ cells into psoriatic lesions, and suppression of circulating CD4+ and CD8+ cells and inflammatory cytokines [92].

*Salvia miltiorrhiza* Bunge also has a long history of use in psoriasis. An in silico study predicted that several compounds from this medicinal plant exert modulatory effects on key pathophysiologies of psoriasis, including inflammation, proliferation, and angiogenesis, via targets such as the apoptosis regulator Bcl-2, caspase-3, TNF, and prostaglandin G/H synthase 2 [93]. Studies on the key compounds of this herb have been conducted, demonstrating the ability of tanshinol to ameliorate IMQ-induced skin lesions and pathological phenotypes in psoriasis mice, and the protective effect of salvianolic acid B against UVB radiation-related skin photoaging [94,95]. Additionally, other studies have reported potential therapeutic effects of *Salvia miltiorrhiza* Bunge’s major constituents, including tanshinone IIA, cryptotanshinone, salvianolic acid B, and rosmarinic acid, on psoriasis, rheumatoid arthritis, and inflammatory bowel diseases through their immunomodulatory activities [96]. 

*Glycyrrhiza glabra* L. is the most widely utilized medicinal plant among EAHM, likely due to its ability to inhibit toxicity in a wide range of combination drugs through its potent antioxidant and anti-inflammatory activities [97]. This herb plays a crucial role in enhancing the safety of EAHM prescriptions as a whole, and its integral use in the vast majority of prescriptions explains why this study did not measure the impact of individual herbs based on frequency analysis alone [98]. The major constituents of *Glycyrrhiza glabra* L., including total flavonoids, glycyrrhizic acid, isoliquiritigenin, 5-(1,1-dimethylallyl)-3,4,4′-trihydroxy-2-methoxy chalcone, licochalcone B, licochalcone A, echinatin, and glycycoumarin, have been shown to inhibit various inflammatory mediators. These include TNF-α, IL-6, inducible nitric oxide synthase, COX-2, NF-κB and MAPK signaling, and prostaglandin E2. Numerous studies have demonstrated the potent anti-inflammatory activity of these compounds, mediated by their action on multiple inflammation-related targets [99]. This evidence strongly suggests that *Glycyrrhiza glabra* L. plays an important role not only in improving safety within EAHM regimens but also in exerting efficacy in modulating the inflammatory pathology of psoriasis. 

*Rehmannia glutinosa* (Gaertn.) DC. Recens is not only a medicinal plant with a remarkably extensive range of indications, including anti-inflammatory, antioxidant, and immunomodulatory properties, but also an EAHM material with the most synergistic combinations with other herbs [100]. Uniquely, the drug is transformed into *Rehmannia glutinosa* (Gaertn.) DC. Preparata after a processing method involving steaming with rice wine nine times, which completely changes its chemical composition, metabolic phenotypes after administration, and targeted diseases [100,101]. In this study, unprocessed *Rehmannia glutinosa* (Gaertn.) DC. was identified as a potent herb, an analysis consistent with the clinical context. Supporting this finding, previous studies have reported that catalpol, the main active ingredient, effectively alleviated skin lesions such as erythema, scaling, and ear thickness in psoriasis in vivo and in vitro models [102]. This effect was mediated by upregulating silent information regulator 1 and suppressing NF-κB and MAPKs signaling pathways. Additionally, several studies have identified that polysaccharides contained within *Rehmannia glutinosa* (Gaertn.) DC. can normalize both non-specific immunity and cellular immunity through the multi-targeted regulation of cytokines, immune molecules, lymphocytes, and NK cells [103].

As demonstrated above, the top-ranked herbs can contribute to improving the efficacy and safety of NB-UVB combination therapy through synergistic effects. These effects are based on multi-component, multi-target mechanisms and appropriate combinations, as supported by previous studies. Additionally, herbs ranked between 6th and 10th—*Scutellaria baicalensis* Georgi, *Paeonia lactiflora* Pall., *Lonicera japonica* Thunb., *Dictamnus dasycarpus* Turcz., and *Arnebia euchroma* I.M.Johnst.—also have a broad base of studies supporting the positive results of the meta-analysis for psoriasis [25,104,105,106,107,108]. Therefore, EAHM individual herbs with the highest PageRank centrality in this study should be considered promising research targets for the development and optimization of improved NB-UVB combination therapies. Furthermore, the network-based approach may warrant further investigation as an additional analysis technique for the unique multidrug environment of EAHM.

## 5. Conclusions

In conclusion, this study found that the application of orally administered EAHM with NB-UVB combination therapy may lead to better efficacy and safety compared to NB-UVB monotherapy. The network meta-analysis showed a relative advantage of IP3 and ZLD in terms of efficacy, as measured by the PASI, and ZYDs and RZC in terms of reduction in AEs incidence. In addition, the RCTs included in this review used other dosage forms, such as granules, pills, and capsules, in addition to decoction, which is the most traditional dosage form for EAHM combination therapy. Therefore, additional studies focusing on adherence and efficacy by dosage form may be able to extend the results of this study. A network analysis across herbs used in individual prescriptions resulted in a ranking of constituent drugs with higher centrality, including *Paeonia suffruticosa* Andrews, *Smilax glabra* Roxb. Rhizoma, *Salvia miltiorrhiza* Bunge, *Glycyrrhiza glabra* L., and *Rehmannia glutinosa* (Gaertn.) DC. Recens. These herbs are predicted to have a greater impact on the above clinical benefits. However, the quality of the evidence is still low due to issues such as sample size, publication bias, and risk of bias, resulting in limitations in the interpretation of the results. Further double-blind, placebo-controlled, multicenter trials are required to reach more robust conclusions. 

## Figures and Tables

**Table 1:** Summary of the certainty of the pairwise meta-analysis rationale.

Intervention and Comparator Intervention	Outcomes	Number of Participants (Studies)	Anticipated Absolute Effects (95% CI)	Quality of the Evidence (GRADE)
EAHM with NB-UVB combined therapy compared NB-UVB monotherapy for psoriasis vulgaris	PASI90	2975 (34)	143 more per 1000 (from 105 more to 186 more)	⨁⨁◯◯ LOW ^a,b^
	PASI60	3249 (38)	213 more per 1000 (from 179 more to 250 more)	⨁⨁◯◯ LOW ^a,b^
	PASI score	2785 (31)	MD 3.3973 PASI score lower (4.2945 lower to 2.5001 lower)	⨁⨁⨁◯ MODERATE ^a^
	AEs incidence rate	1919 (24)	42 fewer per 1000 (from 69 to 9 fewer)	⨁⨁◯◯ LOW ^a,c^
	IL-2	362 (4)	SMD 2.145 SD lower (2.6561 lower to 1.6519 lower)	⨁⨁◯◯ LOW ^a,c^
	IL-17	594 (6)	SMD 1.1598 SD lower (1.8102 lower to 0.5093 lower)	⨁⨁◯◯ LOW ^a,c^
	IL-22	245 (2)	SMD 1.26 SD lower (1.293 lower to 0.7589 lower)	⨁⨁◯◯ LOW ^a,c^
	IL-23	353 (4)	SMD 2.586 SD lower (4.6503 lower to 0.5217 lower)	⨁⨁◯◯ LOW ^a,c^
	TNF-α	306 (3)	SMD 1.1595 SD lower (2.2136 lower to 0.1055 lower)	⨁⨁◯◯ LOW ^a,c^
	INF-γ	362 (4)	SMD 1.9396 SD lower (3.6696 lower to 0.2105 lower)	⨁⨁◯◯ LOW ^a,c^
	CD4+/CD8+ ratio	375 (4)	MD 0.2875 CD4+/CD8+ ratio higher (0.0118 lower to 0.5632 lower)	⨁⨁◯◯ LOW ^a,c^
	DLQI	228 (2)	MD 1.9548 DLQI lower (2.8089 lower to 1.1007 lower)	⨁⨁◯◯ LOW ^a,c^

AEs, adverse events; CD, cluster of differentiation; DLQI, Dermatology Life Quality Index; EAHM, East Asian herbal medicine; INF-γ, interferon gamma; IL, interleukin; MD, mean difference; NB-UVB: narrow band–ultraviolet B therapy; PASI, Psoriasis Area Severity Index; SD, standardized difference; SMD, standardized mean difference; TNF-α, tumor necrosis factor alpha. ^a^ Substantial concerns of risk of bias, ^b^ strongly suspected publication bias, ^c^ confidence intervals overlap less.

**Table 2:** Network characteristics of outcomes included in this Bayesian network meta-analysis.

Characteristic	PASI90	PASI60	PASI Score	AEs Incedence
Number of interventions	28	30	26	21
Number of included trials	34	37	31	24
Total number of patients in network	2975	3249	2785	1919
Total possible pairwise comparisons	378	435	325	210
Total number of pairwise comparisons with direct data	27	29	25	20
Is the network connected?	TRUE	TRUE	TRUE	TRUE
Average outcome (for continuous variables)	Not applicable	Not applicable	6.007	Not applicable
Total number of events in network (for dichotomous variables)	933	2296	Not applicable	284
Number of studies with no zero events	34	37	Not applicable	24
Number of studies with at least one zero events (for dichotomous variables)	0	0	Not applicable	0
Number of studies with all zero events (for dichotomous variables)	0	0	Not applicable	0

## Data Availability

All data employed in this study are included in the manuscript and references. This study utilizes data from the published literature exclusively, and no original data were generated.

## Supplementary Materials

The following supporting information can be downloaded at: https://www.mdpi.com/article/10.3390/nu16162690/s1. Material S1. Description of the search strategy and terms used in each database. Material S2. Summary of studies included in the review. Material S3A. Overview of EAHM prescription name and formulation information for each study. Material S3B. Details of the herbs that constitute the EAHM formulation for each study. Material S4. (A) Forest plot for Gastrointestinal AE in pairwise meta-analysis; (B) Forest plot for Cutaneous AE in pairwise meta-analysis. Material S5. PageRank centrality values and rankings of all EAHM prescription herbs included in this study.
