# Supplementary material for: Efficacy and Safety of Orally Administered East Asian Herbal Medicine Combined with Narrowband Ultraviolet B against Psoriasis: A Bayesian Network Meta-Analysis and Network Analysis
Source: Nutrients. 2024 Aug 13;16(16):2690. doi: 10.3390/nu16162690 (PMC11357435; doi:10.3390/nu16162690)
Supplement: Supplementary file 1 [file nutrients-16-02690-s001.zip › nutrients-3129766-supplementary.pdf]

Efficacy and Safety of Orally Administered East Asian Herbal Medicine Combined with Narrowband Ultraviolet B against Psoriasis: A Bayesian Network Meta-Analysis and Network Analysis

Hee-Geun Jo [1,2,†], Hyehwa Kim [3,†], Eunhye Baek [4], Jihye Seo [5,*] and Donghun Lee [1,*]

[1] Department of Herbal Pharmacology, College of Korean Medicine, Gachon University, 1342 Seongnamdae-ro, Seongnam-si 13120, Republic of Korea; jho3366@hanmail.net
[2] Naturalis Inc., 6, Daewangpangyo-ro, Bundang-gu, Seongnam-si 13549, Republic of Korea
[3] KC Korean Medicine Hospital 12, Haeol 2-gil, Paju-si 10865, Republic of Korea
[4] RexSoft Inc., 1 Gwanak-ro, Seoul 08826, Republic of Korea
[5] Siho Korean Medicine Clinic, 407, Dongtansillicheon-ro, Hwaseong-si 18484, Republic of Korea
* Correspondence: herbabaa@gmail.com (J.S.); dlee@gachon.ac.kr (D.L.)
† These authors contributed equally to this work.

Supplementary Materials

Supplementary Materials S1. Description of the search strategy and terms used in each database.

Supplementary Materials S2. Summary of studies included in the review.

Supplementary Materials S3A. Overview of EAHM prescription name and formulation information for each study.

Supplementary Materials S3B. Details of the herbs that constitute the EAHM formulation for each study.

Supplementary Materials S4. (A) Forest plot for Gastrointestinal AE in pairwise meta-analysis; (B) Forest plot for Cutaneous AE in pairwise meta-analysis.

Supplementary Materials S5. PageRank centrality values and rankings of all EAHM prescription herbs included in this study.

Supplementary Materials S1. Description of the search strategy and terms used in each database.

Pubmed

	Searches	Results
#1	Psoriasis[Mesh]	42404
#2	(Psoriases[Title/Abstract]) OR (Pustulosis of Palms[Title/Abstract] AND Soles[Title/Abstract]) OR (Pustulosis Palmaris et Plantaris[Title/Abstract]) OR (Palmoplantaris Pustulosis[Title/Abstract]) OR (Pustular Psoriasis of Palms[Title/Abstract] AND Soles[Title/Abstract])	188
#3	"Plants, Medicinal"[MeSH] OR "Drugs, Chinese Herbal"[MeSH] OR "Medicine, Chinese Traditional"[MeSH] OR "Medicine, Kampo"[MeSH] OR "Medicine, Korean Traditional"[MeSH] OR "Herbal Medicine"[MeSH] OR "Prescription Drugs"[MeSH] OR "traditional Korean medicine"[Title/abstract] OR "traditional Chinese medicine"[Title/abstract] OR "traditional oriental medicine"[Title/abstract] OR "Kampo medicine"[Title/abstract] OR herb*[Title/abstract] OR decoction*[Title/abstract] OR botanic*[Title/abstract]	234484
#4	#1 AND #2 AND #3	**283**

EMBASE

	Searches	Results
#1	'Psoriasis'/exp	106,080
#2	'Psoriases' OR 'Pustulosis of Palms and Soles' OR 'Pustulosis Palmaris et Plantaris' OR 'Palmoplantaris' OR 'Pustulosis' OR 'Pustular Psoriasis of Palms and Soles'	5857
#3	'medicinal plant'/exp OR 'medicinal plant' OR 'herbaceous agent'/exp OR 'herbaceous agent' OR 'chinese medicine'/exp OR 'chinese medicine' OR 'kampo medicine'/exp OR 'kampo medicine' OR 'kampo medicine (drug)'/exp OR 'kampo medicine (drug)' OR 'korean medicine'/exp OR 'korean medicine' OR 'herbal medicine'/exp OR 'herbal medicine' OR 'prescription drug'/exp OR 'prescription drug' OR 'oriental medicine'/exp OR 'oriental medicine' OR 'alternative medicine'/exp OR 'alternative medicine' OR 'complementary medicine' OR 'herb'/exp OR 'herb' OR 'decoction' OR 'botanic'	585795
#4	#1 AND #2 AND #3	**63**

CENTRAL

	Searches	Results
#1	MeSH descriptor: [Psoriasis] explode all trees	3442
#2	("Psoriases" OR "Pustulosis of Palms and Soles" OR "Palmoplantaris Pustulosis" OR "Pustulosis Palmaris et Plantaris" OR "Pustular Psoriasis of Palms and Soles"):ti,ab,kw	11
#3	MeSH descriptor: [Plants, Medicinal] explode all trees	946
#4	MeSH descriptor: [Drugs, Chinese Herbal] explode all trees	3645
#5	MeSH descriptor: [Medicine, Chinese Traditional] explode all trees	1219

#6	MeSH descriptor: [Medicine, Kampo] explode all trees	46
#7	MeSH descriptor: [Medicine, Korean Traditional] explode all trees	33
#8	MeSH descriptor: [Herbal Medicine] explode all trees	63
#9	MeSH descriptor: [Prescription Drugs] explode all trees	108
#10	("traditional Korean medicine" OR "traditional Chinese medicine" OR "Traditional oriental medicine" OR "Kampo medicine" OR herb* OR decoction* OR botanic*):ti,ab,kw	18819
#11	(#1 OR #2) AND (#3 OR #4 OR #5 OR #6 OR #7 OR #8 OR #9 OR #10) in Trials	**48**

OASIS

	Searches	Results
#1	건선 AND 한약	**5**

KISS

	Searches	Results
#1	건선 AND 한약	**3**

RISS

	Searches	Results
#1	건선 AND 한약	**2**

KCI

	Searches	Results
#1	건선 AND 한약	**7**

CNKI

	Searches	Results
#1	(TI='银屑病' OR '牛皮癣' OR '白疕' OR '寻常型银屑病' OR '点滴型银屑病' OR +'银屑病关节炎' OR '脓疱型银屑病' OR '关节病型银屑病') AND (TI='中药' OR '中医药' OR '中草药' OR '本草' OR '汤' OR '丸' OR '散' OR '方' OR '颗粒 OR '胶囊' OR '自拟') AND (AB='银屑病' OR '牛皮癣' OR '白疕' OR '寻常型银屑病' OR '点滴型银屑病' OR '银屑病关节炎' OR '脓疱型银屑病' OR '关节病型银屑病') AND (AB='中药' OR '中医药' OR '中草药' OR '本草' OR '汤' OR '丸' OR '散' OR '方' OR '颗粒 OR '胶囊' OR '自拟') AND (AB='随机')	**1161**

Wanfang data

	Searches	Results
#1	题名:("银屑病"or"牛皮癣"or"白疕"or"寻常型银屑病"or"点滴型银屑病"or "银屑病关节炎"or "脓疱型银屑病"or "关节病型银屑病") and 题名:("汤" or "丸" or "散" or "中药") and 摘要:("银屑病"or"牛皮癣"or"白疕"or"寻常型银屑病"or"点滴型	**813**

	银屑病”or “银屑病关节炎”or ”脓疱型银屑病”or ”关节病型银屑病”) and 摘要:(" 汤” or “丸” or “散" or "中药") and 摘要:("随机")	

CiNii

	Searches	Results
#1	(“銀屑” OR “Psoriasis” OR “Pustulosis of Palms and Soles” OR “Palmoplantaris Pustulosis” OR “Pustulosis Palmaris et Plantaris” OR “Pustular Psoriasis of Palms and Soles”) AND (“traditional Korean medicine” OR “traditional Chinese medicine” OR “Traditional oriental medicine” OR “Kampo medicine” OR herb OR decoction OR botanic OR 漢方薬 OR ハーブ OR 散 OR 汤 OR 丸)	**49**

Supplementary Materials S2. Summary of studies included in the review.

Included Study (Reference)	Trial Design	Number of Patients (Male/Female); Age (Mean ± SD)		Interventions		Outcome Index (Intergroup Differencies p-Value)	Treatment period	Adverse Events (Case/Symptom)
		Trial	*Control*	*Trial*	*Control*			
Zhong 2007[1]	Randomized(by random number table); Single center; Parallel	42 (28/14)<br>34.55± 7.18 y	42 (30/12)<br>33.79 ± 4.25 y	1. Xiaoyin Granules1<br>(b.i.d.)<br>2. NB-UVB (Wavelength range 310 -350 nm, initial dose 0.3 -0.5 J/cm², Cumulative maximum dose 2.2 J/cm², t.i.w.)	1. NB-UVB (Wavelength range 310 -350 nm, initial dose 0.3 -0.5 J/cm², Cumulative maximum dose 2.2 J/cm², t.i.w.)	1. PASI 60 ($p < 0.05$)<br>2. PASI score ($p < 0.05$)	8 w	Trial: 12 AEs<br>Control: NR<br>Including xerostomia, gastric discomfort, nausea and loose stool
Lu 2009[2]	Randomized(by random number table); Single center; Parallel	36 (22/14)<br>32.6 y (17–46 y)	32 (18/14)<br>35.1 y (18–69 y)	1. Keyin Pill (b.i.d.)<br>2. NB-UVB (Wavelength range 311 -315 nm, initial 0.4 - 0.5 J/cm², Cumulative maximum dose 2.5 J/cm², b.i.w.)	1. NB-UVB (Wavelength range 311 -315 nm, initial 0.4 - 0.5 J/cm², Cumulative maximum dose 2.5 J/cm², b.i.w.)	1. PASI 90 ($p < 0.05$)<br>2. PASI 60 ($p < 0.05$)<br>3. PASI score ($p < 0.05$)	8 w	Trial: 7 AEs<br>(4 cutaneous erythema and burning sensations, 3 transient elevated liver enzymes)<br>Control: 8 AEs<br>(8 cutaneous erythema and burning sensations,)
Lu 2010[3]	Randomized(by simple randomization);	Both group<br>156 (92/64)	Both group<br>156 (92/64)	1. Investigator Presciption1 (b.i.d.)	1. NB-UVB (initial 0.3 J/cm², Cumulative maximum dose	1. PASI 90 ($p < 0.05$)	8 w	Trial: 0 AEs<br>Control: 0 AEs

	Single center; Parallel	34.5 y (18-65 y)	34.5 y (18-65 y)	2. NB-UVB (initial 0.3 J/cm^2, Cumulative maximum dose 2.0 J/cm^2, q.o.d.)	2.0 J/cm^2, q.o.d.)	2. PASI 60 ($p < 0.05$)<br>3. PASI score ($p < 0.05$)		
Gu 2011[4]	Randomized; Single center; Parallel	42 (27/15)<br>35.5 y (12–69 y)	30 (18/12)<br>32.3 y (10–67 y)	1. Xiaofeng Granules (t.i.d.)<br>2. NB-UVB (Wavelength range 311 -313 nm, initial 0.3 - 0.5 J/cm^2, b.i.w.)	1. Immunosuppressant (compound amino peptide tablets 5t, b.i.d.)	1. PASI 90 ($p < 0.05$)<br>2. PASI 60 ($p < 0.05$)	8 w	Trial: 0 AEs<br>Control: 0 AEs<br>Detailed information NR
Liu 2011[5]	Randomized(by simple randomization); Single center; Parallel	Both group<br>122 (78/44)<br>(12–60 y)	Both group<br>122 (78/44)<br>(12–60 y)	1. Xiaoyin Granules2(t.i.d.)<br>2. NB-UVB (Wavelength range 310 -315 nm, initial 0.3 J/cm^2, Cumulative maximum dose 3 J/cm^2, b.i.w.)	1. NB-UVB (Wavelength range 310 -315 nm, initial 0.3 J/cm^2, Cumulative maximum dose 3 J/cm^2, b.i.w.)	1. PASI 90 ($p < 0.05$)<br>2. PASI 60 ($p < 0.05$)<br>3. PASI score ($p < 0.05$)	8 w	Trial: 17 AEs<br>(9 burning sensations, 8 NB-UVB irradiation site skin pruritus)<br>Control: 15 AEs<br>(8 burning sensations, 7 NB-UVB irradiation site skin pruritus)
Pang 2011[6]	Randomized; Single center; Parallel	38 (17/21)<br>23.41±6.18 y	30 (18/12)<br>27.41±7.12 y	1. Total Glucosides of Paeony Capsules (t.i.d.)<br>2. NB-UVB (Wavelength range 311±3nm, q.o.w.)	1. NB-UVB (Wavelength range 311±3nm, q.o.w.)	1. PASI 90 ($p < 0.05$)<br>2. PASI 60 ($p < 0.05$)	60 d	Trial: 3 AEs (2 diarrhea with abdominal discomfort, 1 skin pruritus)<br>Control: 2 AEs<br>(2 xeroderma with skin pruritus)

Xia 2011[7]	Randomized; Single center; Parallel	40 (25/15)<br>35.2 y (16–58 y)	40(21/19)<br>34.5 y (3–23 y)	1. Liangxue Xiaofeng Decoction (b.i.d.)<br>2. NB-UVB (Wavelength range 311 -313 nm, initial 0.3 J/cm², b.i.w.)	1. NB-UVB (Wavelength range 311 -313 nm, initial 0.1 J/cm², b.i.w.)	1. PASI 90 ($p < 0.05$)<br>2. PASI 60 ($p < 0.05$)	8 w	Trial: 5 AEs<br>(5 NB-UVB irradiation site skin pruritus with erythema)<br>Control: 5 AEs<br>(5 NB-UVB irradiation site skin pruritus with erythema)
Xu 2011[8]	Randomized; Single center; Parallel	30 (19/11)<br>32.24±10.77 y	25 (16/9)<br>31.74±11.71 y	1. Investigator Presciption2 (b.i.d.)<br>2. NB-UVB (Wavelength range 310 -315 nm, initial 0.4 J/cm², b.i.w.)	1. NB-UVB (Wavelength range 310 -315 nm, initial 0.4 J/cm², b.i.w.)	1. PASI 90 ($p < 0.05$)<br>2. PASI 60 ($p < 0.05$)<br>3. PASI score ($p < 0.05$)	8 w	Trial and Control: 31 AEs<br>(4 Mild diarrhea after taking oral medications, 7 NB-UVB irradiation site erhythema, 20 NB-UVB irradiation site skin pruritus)
Zhang 2011[9]	Randomized; Single center; Parallel	30 (23/7)<br>21.22±11.54 y	30 (22/8)<br>24.13±9.34 y	1. Liangxue Xiaobian Decoction (b.i.d.)<br>2. NB-UVB (Wavelength range 311 nm, initial 0.4-0.5 J/cm², t.i.w.)	1. NB-UVB (Wavelength range 311 nm, initial 0.4-0.5 J/cm², t.i.w.)	1. PASI 70 ($p < 0.05$)	8 w	Trial: 4 AEs<br>(4 NB-UVB irradiation site skin pruritus with erythema and xeroderma)<br>Control: 5 AEs<br>(5 NB-UVB irradiation site skin pruritus with erythema and xeroderma)
Chen 2012[10]	Randomized; Single center; Parallel	30 (18/12)<br>32.1±6.6 y	30 (16/14)<br>38.4±5.9 y	1. Qingri Jiedu Huoxue Decoction (b.i.d.)	1. NB-UVB (initial 0.5 J/cm², q.o.d.)	1. PASI 90 ($p < 0.05$)	4 w	Trial: 6 AEs<br>(6 mild diarrhea)<br>Control: 9AEs

				2. NB-UVB (initial 0.5 J/cm², q.o.d.)		2. PASI 60 (p < 0.05)<br>3. PASI score (p < 0.05)		(9 NB-UVB irradiation site skin erythema and burning sensation)
Li 2012[11]	Randomized; Single center; Parallel	36 (20/16)<br>33.5±2.0 y	36 (20/16)<br>35.0±1.0 y	1. Investigator Presciption3 (b.i.d.)<br>2. NB-UVB (initial 0.3-0.5 J/cm², b.i.w.)	1. NB-UVB (initial 0.3-0.5 J/cm², q.o.d.)	1. PASI 90 (p < 0.05)<br>2. PASI 60 (p < 0.05)<br>3. PASI score (p < 0.05)	8 w	NR
Han 2013[12]	Randomized; Single center; Parallel	40 (20/20)<br>32.95±2.60 y	40 (19/21)<br>33.45±2.50 y	1. Investigator Presciption3 (b.i.d.)<br>2. NB-UVB (initial 0.3-0.5 J/cm², b.i.w.)	2. NB-UVB (initial 0.3-0.5 J/cm², q.o.d.)	1. PASI 90 (p < 0.05)<br>2. PASI 60 (p < 0.05)<br>3. PASI score (p < 0.05)	8 w	NR
Wang 2013[13]	Randomized; Single center; Parallel	60 (35/25)<br>33.65±8.77 y	60 (38/22)<br>35.37±11.36 y	1.Yinxie Capsule<br>(t.i.d.)<br>2. NB-UVB (Wavelength	2. NB-UVB (Wavelength range 309 -313 nm, t.i.w.)	1. PASI 90 (p < 0.05)<br>2. PASI 60 (p < 0.05)	12 w	Trial: 4 AEs<br>(2 NB-UVB irradiation site skin erythema, 1 skin pruritus, 1 mild diarrhea)<br>Control: 5AEs

				range 309 -313 nm, t.i.w.)		3. PASI score ($p < 0.05$)		(2 NB-UVB irradiation site skin erythema, 3 skin pruritus)
Yin 2013[14]	Randomized(by random number table); Single center; Parallel	50 (24/26)<br>34.66±10.45 y	50 (25/25)<br>33.64±9.83 y	1.Zhuhuang Granules<br>(t.i.d.)<br>2. NB-UVB (Wavelength range 310 -315 nm, initial 0.3 - 0.5 J/cm^2, b.i.w.)	1. NB-UVB (Wavelength range 310 -315 nm, initial 0.3 - 0.5 J/cm^2, b.i.w.)	1. PASI 90 ($p < 0.05$)<br>2. PASI 60 ($p < 0.05$)<br>3. PASI score ($p < 0.05$)	8 w	Trial: 3 AEs<br>(2 NB-UVB irradiation site skin erythema, 1 burning sensation)<br>Control: 6 AEs<br>(2 NB-UVB irradiation site skin erythema, 1 burning sensation, 3 skin pruritus)
Yu 2014[15]	Randomized(by simple randomization); Single center; Parallel	Both group<br>86 (NR)<br>(16-65 y)	Both group<br>86 (NR)<br>(16-65 y)	1.Tuiyin Decoction<br>(b.i.d.)<br>2. NB-UVB (Wavelength range 311 nm, initial 0.5 J/cm^2, b.i.w.)	1. NB-UVB (Wavelength range 310 -315 nm, initial 0.3 - 0.5 J/cm^2, b.i.w.)	1. PASI 90 ($p < 0.05$)<br>2. PASI 60 ($p < 0.05$)<br>3. PASI score ($p < 0.05$)	12 w	Trial: 6 AEs<br>(2 burning sensation, 2 skin pruritus, 2 mild diarrhea)<br>Control: 7 AEs<br>(3 burning sensation, 2 stabbing pain, 2 skin pruritus)
Zhang 2014[16]	Randomized; Single center; Parallel	60 (35/25)<br>35.97±12.41 y	40 (22/18)<br>35.78±13.78 y	1.Liangxue Xiaofeng Decoction<br>(b.i.d.)<br>2. NB-UVB (initial 0.2 -0.5 J/cm^2, b.i.w.)	1. NB-UVB (initial 0.2 -0.5 J/cm^2, b.i.w.)	1. PASI 90 ($p < 0.05$)<br>2. PASI 60 ($p < 0.05$)	8 w	NR

						3. PASI score ($p < 0.05$)<br>4. IL-2 ($p < 0.05$)<br>5. IFN-γ ($p < 0.05$)		
Cheng 2015[17]	Randomized; Single center; Parallel	36 (23/13)<br>26±6.2 y	36 (25/11)<br>27±5.6 y	1.Qingre Liangxue Decoction (t.i.d.)<br>2. NB-UVB (Wavelength range 311 nm, initial 0.3 J/cm², q.o.d.)	1. NB-UVB (Wavelength range 311 nm, initial 0.3 J/cm², q.o.d.)	1. PASI 90 ($p < 0.05$)<br>2. PASI 60 ($p < 0.05$)	4 w	Trial: 5 AEs<br>(burning sensation, skin erythematous, and skin pruritus)<br>Control: 6 AEs<br>(burning sensation, skin erythematous, and skin pruritus)
Hu 2015[18]	Randomized; Single center; Parallel	58 (47/17)<br>34.7±8.9 y	58 (39/19)<br>34.1±8.3 y	1. Zidan Yinxie Granules(t.i.d.)<br>2. NB-UVB (Wavelength range 311 nm, initial 0.3-0.5 J/cm², t.i.w.)	1. NB-UVB (Wavelength range 311 nm, initial 0.3-0.5 J/cm², t.i.w.)	1. PASI score ($p < 0.05$)<br>2. DLQI ($p < 0.05$)<br>3. IL-17 ($p < 0.05$)<br>4. IL-22 ($p < 0.05$)	8 w	NR

						5. IL-23 ($p < 0.05$)		
Pang 2015[19]	Randomized; Single center; Parallel	40 (23/17)<br>27.8 y (14-66 y)	40 (19/21)<br>30.5 y (14-62 y)	1. Shufeng Jiedu Capsules (t.i.d.)<br>2. NB-UVB (initial 0.7 J/cm², b.i.w.)	1. NB-UVB (initial 0.7 J/cm², b.i.w.)	1. PASI 90 ($p < 0.05$)<br>2. PASI 60 ($p < 0.05$)<br>3. PASI score ($p < 0.05$)	8 w	NR
Zheng 2015[20]	Randomized; Single center; Parallel	Both group<br>60 (33/27)<br>(18-53 y)	Both group<br>60 (33/27)<br>(18-53 y)	1. Runzao Zhiyang Capsule (t.i.d.)<br>2. NB-UVB (Wavelength range 310-350 nm, initial 0.6 J/cm², Cumulative maximum dose 3.0 J/cm², q.o.d.)	1. NB-UVB (Wavelength range 310-350 nm, initial 0.6 J/cm², Cumulative maximum dose 3.0 J/cm², q.o.d.)	1. PASI 90 ($p < 0.05$)<br>2. PASI 60 ($p < 0.05$)<br>3. CD4+/CD8+ ($p < 0.01$)	8 w	Trial: 1 AEs<br>(1 mild diarrhea)<br>Control: 5 AEs<br>(2 burning sensation, 3 skin erythematous with pruritus)
Zou 2015[21]	Randomized; Single center; Parallel	Both group<br>76 (42/34)	Both group<br>76 (42/34)	1. Total Glucosides of	1. NB-UVB (Wavelength range 310-350 nm, initial 0.3-0.5 J/cm²,	1. PASI 90 ($p < 0.05$)	12 w	Trial: 7 AEs<br>(5 xeroderma, 2 nausea)<br>Control: 8 AEs

		(37.75±5.61 y)	(37.75±5.61 y)	Paeony Capsules (t.i.d.)<br>2. NB-UVB (Wavelength range 311 nm, initial 0.3-0.5 J/cm^2, Cumulative maximum dose 3.0 J/cm^2, t.i.w.)	Cumulative maximum dose 3.0 J/cm^2, t.i.w.)	2. PASI 60 ($p < 0.05$)<br>3. PASI score ($p < 0.05$)		(8 skin pruritus with scale)
Li 2016[22]	Randomized(by simple randomization); Single center; Parallel	30 (19/11)<br>45.23±15.52 y	30 (14/16)<br>42.51±09.52 y	1. Total Glucosides of Paeony Capsules (t.i.d.)<br>2. NB-UVB (Wavelength range 310-315 nm, initial 0.3-0.4 J/cm^2, t.i.w.)	1. NB-UVB (Wavelength range 310-315 nm, initial 0.3-0.4 J/cm^2, t.i.w.)	1. PASI 90 ($p < 0.05$)<br>2. PASI 60 ($p < 0.05$)<br>3. PASI score ($p < 0.001$)<br>4. CD4+/CD8+ ($p < 0.05$)	8 w	NR
Liu 2016[23]	Randomized; Single center; Parallel	100 (53/47)<br>35.8±6.7 y	100 (56/44)<br>34.7±7.1 y	1. Ziyin Liangxue Decoction (b.i.d.)<br>2. NB-UVB (initial 0.5 J/cm^2, b.i.w.)	1. NB-UVB (initial 0.5 J/cm^2, b.i.w.)	1. PASI 90 ($p < 0.05$)<br>2. PASI 60 ($p < 0.05$)	8 w	NR

						3. PASI score ($p < 0.05$)		
Song 2016[24]	Randomized; Single center; Parallel	30 (16/14)<br>31.4±4.7 y	30 (15/15)<br>32.4±5.6 y	1. Xiaoyin Decoction<br>2. NB-UVB (Dose details not reported, t.i.w.)	1. NB-UVB (Dose details not reported, t.i.w.)	1. PASI 90 ($p < 0.05$)<br>2. PASI 60 ($p < 0.05$)<br>3. PASI score ($p < 0.01$)<br>4. IL-17 ($p < 0.05$)<br>5. IL-23 ($p < 0.05$)<br>6. IL-6 ($p < 0.05$)	8 w	Trial: 3 AEs<br>(3 mild diarrhea)<br>Control: 2 AEs<br>(2 NB-UVB irradiation site skin erythema with pruritus)
Wu 2016[25]	Randomized(by random number table); Single center; Parallel	29 (15/14)<br>32.91±7.52 y	30 (14/16)<br>33.1±7.2 y	1. Runzao Zhiyang Capsule<br>(t.i.d.)<br>2. NB-UVB (initial 0.5 J/cm^2,	1. NB-UVB (initial 0.5 J/cm^2, Cumulative maximum dose 3.0 J/cm^2, b.i.w.)	1. PASI 90 ($p < 0.05$)<br>2. PASI 60 ($p < 0.05$)	8 w	Trial: 2 AEs<br>(2 mild diarrhea)<br>Control: 3 AEs<br>(2 NB-UVB irradiation site skin erythema with burning sensation)

				Cumulative maximum dose 3.0 J/cm², b.i.w.)		3. PASI score (p < 0.01)		
Zhou 2016[26]	Randomized; Single center; Parallel	31 (17/14)<br>35.3 y (18-62 y)	27 (16/11)<br>34.7 y (21-57 y)	1. Runzao Zhiyang Capsule (t.i.d.)<br>2. NB-UVB (Wavelength range 309-311 nm, t.i.w.)	1. NB-UVB (Wavelength range 309-311 nm, t.i.w.)	1. PASI 90 (p < 0.05)<br>2. PASI 60 (p < 0.05)<br>3. PASI score (p < 0.01)	8 w	Trial: 3 AEs<br>(2 skin erythema, 1 skin scale)<br>Control: 9 AEs<br>(4 skin erythema, 2 skin pruritus, 3 skin scale)
Zhu 2016[27]	Randomized(by random number table); Single center; Parallel	40 (23/17)<br>27.69±5.36 y	40 (22/18)<br>28.74±5.22 y	1. Xiaoyin Granules3 (t.i.d.)<br>2. NB-UVB (Wavelength range 311 nm, initial 0.4 J/cm², q.o.d.)	1. NB-UVB (Wavelength range 311 nm, initial 0.4 J/cm², q.o.d.)	1. PASI 90 (p < 0.05)<br>2. PASI 60 (p < 0.05)<br>3. PASI score (p < 0.01)<br>4. IL-17 (p < 0.01)<br>5. IL-23 (p < 0.01)	8 w	Trial: 3 AEs<br>(1 mild burning sensation, 1 mild skin pruritus, 1 mild skin erythema)<br>Control: 7 AEs<br>(3 mild burning sensation, 3 mild skin pruritus, 1 mild skin erythema)

						6. TNF-α ($p < 0.01$)		
Ji 2017[28]	Randomized(by random number table); Single center; Parallel	41 (29/12)<br>44.02±5.15 y	41 (27/14)<br>44.15±5.71 y	1. Zidan Yinxie Granules (t.i.d.)<br>2. NB-UVB (Wavelength range 311 nm, initial 0.15 J/cm², Cumulative maximum dose 0.8 J/cm², t.i.w.)	1. NB-UVB (Wavelength range 311 nm, initial 0.15 J/cm², Cumulative maximum dose 0.8 J/cm², t.i.w.)	1. PASI 90 ($p < 0.05$)<br>2. PASI 60 ($p < 0.05$)<br>3. PASI score ($p < 0.01$)	12 w	Trial: 1 AE<br>(1 xeroderma with pruritus)<br>Control: 8 AEs<br>(1 nausea, 6 xeroderma with pruritus, 1 dizziness)
Li 2017[29]	Randomized(by random number table); Single center; Parallel	64 (37/27)<br>48.64±8.63 y	64 (38/26)<br>49.15±9.26 y	1. Biejia Jian Pills (t.i.d.)<br>2. NB-UVB (Wavelength range 311 nm, initial 0.3-0.5 J/cm², t.i.w.)	2. NB-UVB (Wavelength range 311 nm, initial 0.3-0.5 J/cm², t.i.w.)	1. PASI 90 ($p < 0.05$)<br>2. PASI 60 ($p < 0.05$)<br>3. PASI score ($p < 0.01$)<br>4. CD4+/CD8+ ($p < 0.05$)	12 w	Trial: 5 AEs<br>(2 xeroderma, 1 skin pruritus, 2 burning sensation)<br>Control: 13AEs<br>(5 xeroderma, 2 skin pruritus, 6 burning sensation)
Lin 2017[30]	Randomized(by random	67 (39/28)	62 (37/25)	1.	1. NB-UVB (Dose details	1. PASI 90 ($p < 0.05$)	8 w	NR

	number table); Single center; Parallel	39.28±10.21 y	40.18±11.83 y	Total Glucosides of Paeony Capsules (t.i.d.)<br>2. NB-UVB (Dose details not reported, t.i.w.)	not reported, t.i.w.)	2. PASI 60 ($p < 0.05$)<br>3. IL-17 ($p < 0.05$)<br>4. IL-22 ($p < 0.05$)<br>5. TNF-α ($p < 0.05$)		
Sun 2017[31]	Randomized; Single center; Parallel	Both group<br>50 (NR)<br>47y (18-70 y)	Both group<br>50 (NR)<br>47y (18-70 y)	1.<br>Bai Bi Decoction (b.i.d.)<br>2. NB-UVB (Dose details not reported, t.i.w.)	1. NB-UVB (Dose details not reported, t.i.w.)	1. PASI 90 ($p < 0.05$)<br>2. PASI 60 ($p < 0.05$)	12 w	NR
Xie 2017[32]	Randomized; Single center; Parallel	49 (34/15)<br>38.1±5.2 y	48 (32/16)<br>37.6±5.4 y	1.<br>Compound Qingdai Capsule (t.i.d.)<br>2. NB-UVB (Wavelength range 308 nm,	2. NB-UVB (Wavelength range 308 nm, initial 0.7 J/cm^2, b.i.d.)	1. PASI 90 ($p < 0.01$)<br>2. PASI 60 ($p < 0.01$)	6 w	Trial: 8 AEs<br>(3 skin pigmentation, 3 burning sensation, 2 gastric discomfort with nausea)<br>Control: 7 AEs<br>(3 skin pigmentation, 4 burning sensation)

				initial 0.7 J/cm², b.i.d.)		3. IL-17 (p < 0.01)<br>4. IL-22 (p < 0.01)<br>5. TNF-α (p < 0.01)		
Shi 2018[33]	Randomized; Single center; Parallel	45(24/21)<br>35.48±6.33 y	45(23/22)<br>35.12±6.45 y	1. Liangxue Zhiyang Decoction (b.i.d.)<br>2. NB-UVB (initial 0.3 J/cm², b.i.w.)	2. NB-UVB (initial 0.3 J/cm², b.i.w.)	1. PASI 90 (p < 0.01)<br>2. PASI 60 (p < 0.05)<br>3. PASI score (p < 0.01)<br>4. IL-2 (p < 0.05)<br>5. IFN-γ (p < 0.05)	8 w	Trial: no SAE<br>Control: no SAE<br>(detailed AE information NR)
Xue 2018[34]	Randomized; Single center; Parallel	30(16/14)<br>31.4±4.7 y	30(15/15)<br>32.4±5.6 y	1. Xiaoyin Decoction (t.i.d.)	1. NB-UVB (Dose details not reported, t.i.w.)	1. PASI 90 (p < 0.05)	8 w	Trial: 3 AEs<br>(3 mild diarrhea)<br>Control: 2 AEs

				2. NB-UVB (Dose details not reported, t.i.w.)		2. PASI 60 ($p < 0.05$)<br>3. PASI score ($p < 0.01$)<br>4. IL-2 ($p < 0.01$)<br>5. IL-4 ($p < 0.01$)<br>5. IL-10 ($p < 0.01$)<br>5. IFN-γ ($p < 0.05$)		(2 skin erythema with pruritus)
Mao 2019[35]	Randomized(by random number table); Single center; Parallel	48(31/17)<br>31.35±15.72 y	48(28/20)<br>35.24±12.35 y	1. Liangxue Jiedu Decoction (t.i.d.)<br>2. NB-UVB (Dose details not reported, t.i.w.)	1. NB-UVB (Dose details not reported, t.i.w.)	1. PASI 90 ($p < 0.05$)<br>2. PASI 60 ($p < 0.05$)<br>3. PASI score ($p < 0.05$)	4 w	Trial: 4 AEs<br>(4 mild diarrhea)<br>Control: 2 AEs<br>(2 mild skin pruritus)

Meng 2020[36]	Randomized; Single center; Parallel	40(24/16)<br>38.7±9.4 y	39(25/14)<br>40.3±8.6 y	1. Modified Liangxue Xiaoyin Decoction (b.i.d.)<br>2. NB-UVB (initial 0.5-0.7 J/cm², Cumulative maximum dose 4 J/cm², t.i.w.)	1. NB-UVB (initial 0.5-0.7 J/cm², Cumulative maximum dose 4 J/cm², t.i.w.)	1. PASI 90 ($p < 0.05$)<br>2. PASI 60 ($p < 0.05$)<br>3. PASI score ($p < 0.01$)	8 w	Trial: 5 AEs<br>(2 mild abdominal distention, 3 frequent defecation)<br>Control: 4 AEs<br>(4 aggravated skin pruritus)
Liu 2021[37]	Randomized; Single center; Parallel	48(27/21)<br>41.22±3.71 y	48(25/23)<br>41.35±3.42 y	1. Liangxue Shufeng Decoction (b.i.d.)<br>2. NB-UVB (Wavelength range 311 nm, initial 0.4 J/cm², Cumulative maximum dose 1.5 J/cm², t.i.w.)	1. NB-UVB (Wavelength range 311 nm, initial 0.4 J/cm², Cumulative maximum dose 1.5 J/cm², t.i.w.)	1. PASI score ($p < 0.05$)	12 w	Trial: no SAE<br>Control: no SAE<br>(detailed AE information NR)
Guo 2021[38]	Randomized(by random number table); Single center; Parallel	40(21/19)<br>39.95±9.63 y	40(23/17)<br>41.23±9.99 y	1. Huaixiang Wushe Granule (b.i.d.)<br>2. NB-UVB (initial 0.5-0.7 J/cm², Cumulative	1. NB-UVB (initial 0.5-0.7 J/cm², Cumulative maximum dose 4 J/cm², t.i.w.)	1. PASI 90 ($p < 0.05$)<br>2. PASI 60 ($p < 0.05$)	8 w	NR

				maximum dose 4 J/cm², t.i.w.)		3. PASI score ($p < 0.01$)<br><br>4. CD4+/CD8+ ($p < 0.05$)		
Lu 2021[39]	Randomized; Single center; Parallel	64(38/26)<br><br>13.21±3.92 y	40(24/16)<br><br>12.86±3.47 y	1. Xiaofeng San (b.i.d.)<br><br>2. NB-UVB (Wavelength range 311-313 nm, Cumulative maximum dose 2.8 J/cm², t.i.w.)	1. NB-UVB (Wavelength range 311-313 nm, Cumulative maximum dose 2.8 J/cm², t.i.w.)	1. PASI 90 ($p < 0.01$)<br><br>2. PASI 60 ($p < 0.01$)<br><br>3. PASI score ($p < 0.01$)	24 w	Trial: 15 AEs<br><br>(9 skin pruritus with scale, 6 skin erythema)<br><br>Control: 10 AEs<br><br>(7 skin erythema with scale, 3 skin discoloration)
Liu 2022[40]	Randomized(by random number table); Single center; Parallel	56(33/23)<br><br>38.22±8.54 y	56(36/20)<br><br>36.95±8.40 y	1. Huoxue Jiedu Decoction (t.i.d.)<br><br>2. NB-UVB (Wavelength range 311 nm, initial 0.5 J/cm², Cumulative maximum dose 2.5 J/cm², q.o.d.)	1. NB-UVB (Wavelength range 311 nm, initial 0.5J/cm², Cumulative maximum dose 2.5 J/cm², q.o.d.)	1. PASI score ($p < 0.01$)<br><br>2. IL-2 ($p < 0.01$)<br><br>3. IL-4 ($p < 0.01$)<br><br>4. IL-10 ($p < 0.01$)	8 w	Trial: 6 AEs<br><br>(2 xeroma and xerostomia, 1 gastrointestinal discomfort, 3 mild skin pruritus)<br><br>Control: 10 AEs<br><br>(4 xeroma and xerostomia, 2 gastrointestinal discomfort, 1 mild burning sensation, 1 mild skin erythema, 2 mild skin pruritus)

5. IL-17 ($p < 0.01$)
6. IFN-γ ($p < 0.01$)
7. CD4+/CD8+ ($p < 0.01$)

AEs: adverse events; b.i.d: bis in die; b.i.w: bis in week; c: capsules; d: days; CD: cluster of differentiation; DLQI: dermatology life quality index; g: grams; IFN: interferon; IL: interleukin; m: months; mg: milligrams; J:joule;NB-UVB: narrow band ultar violet B phtotherapy; NR: not reported; PASI: psoriasis area severity index; q.d: quaque die ; SAE: serious adverse events; SD: standard deviation ; t: tablets ; t.i.d: ter in die ; t.i.w: ter in week; TNF: tumor necrosis factor; w: weeks ; y: years ; μg: micrograms.

References

1. Zhong, J.; Lu, C.; He, W.; Liang, Y.; Huen, G. Narrow-Band Medium-Wave Ultraviolet Rays Combined with "Xiaoyin Granules" Treat 42 Cases of Psoriasis of Blood Deficiency and Wind-Dryness Type. *South. China J. Derm.-Venereol.* **2007**, *14*, 98–100.
2. Lu, P. Clinical Observation of Keyin Pill Combined with Narrow-Band Ultraviolet B for the Treatment of Psoriasis Vulgaris. *Chin. J. Dermatovenereology* **2009**, *23*, 539–540.
3. Lu, X.; Gan, C.; Tian, C. 78 Cases of Psoriasis Treated with Traditional Chinese Medicine Combined with Narrow-Spectrum Medium-Wave Ultraviolet Irradiation. *Guide China Med.* **2010**, *8*, 115–116, doi:10.15912/j.cnki.gocm.2010.04.100.
4. Gu, S.; Sang, Z. Xiaofeng Granules Combined with Narrow-Band Medium-Wave Ultraviolet Irradiation in the Treatment of 42 Cases of Psoriasis Vulgaris. *Henan Tradit. Chin. Med.* **2011**, *31*, 1310–1311, doi:10.16367/j.issn.1003-5028.2011.11.079.
5. Liu, Y.; Yan, L. Clinical Observation on Combination of Xiaoyin Granules with NB-UVB for Reducing Relapse Rate of Psoriasis. *J. Clin. Dermatol.* **2011**, *40*, 767–769.
6. Pang, L. Observation of Efficacy of TGP Capsule Combined with Narrow Band Ultraviolet（NB-UVB） Irradiation in the Treatment of Psoriasis Vulgaris. *China J. Chin. Med.* **2011**, *27*, 223–224, doi:10.16368/j.issn.1674-8999.2011.02.056.
7. Xia, X.; Zhang, W. Observation on the Efficacy of Liangxue Xiaofeng Decoction Combined with Narrow-Band Ultraviolet Rays in the Treatment of Psoriasis Vulgaris. *J. Pract. Tradit. Chin. Med.* **2011**, *27*, 371–372.
8. Xu, P.; Li, H. Observation on the Efficacy of Traditional Chinese Medicine Combined with Narrow-Band Ultraviolet Light in the Treatment of Psoriasis Vulgaris. *J. New Chin. Med.* **2011**, *43*, 66–68, doi:10.13457/j.cnki.jncm.2011.10.087.
9. Zhang, T. Clinical Observation on 30 Cases of Psoriasis Treated with Nbuvb Combined with Liangxue Xiaobian Decoction. *Med. Inf.* **2011**, *6*, 3412–3413, doi:10.3969/j.issn.1006-1959.2011.07.644.

10. Chen, D. The Effect of Modified Qingrijieduhuoxue Soup Combined with NB-UVB on 30 Cases of Psoriasis Vulgaris. *Chin. J. Aesthetic Med.* **2012**, *21*, 309, doi:10.15909/j.cnki.cn61-1347/r.2012.14.520.
11. Li, F. Ultraviolet Combined with Traditional Chinese Medicine for Treating Blood-Deficiency Wind-Attack Psoriasis Randomized Controlled Observation. *J. Pract. Tradit. Chin. Intern. Med.* **2012**, *26*, 67–68.
12. Han, L.; Zhao, X.; Zhang, H.; Xie, L.; Liu, Y.; Zhang, S. Ultraviolet Rays Combined with Oral Administration of Traditional Chinese Medicine in the Treatment of Blood Deficiency and Wind Dryness Type Psoriasis: Observation on the Efficacy of 40 Cases. *J. Guiyang Coll. Tradit. Chin. Med.* **2013**, *35*, 74–75, doi:10.3969/j.issn.1002-1108.2013.04.0035.
13. Wang, Y.; Zhao, J.; Ding, Q.; Liu, C. Curative Effect Observation of Yin-Xie Capsule Combined with Narrow Band Ultraviolet B in the Treatment of Psoriasis Vulgaris. *Core Yin-Xie Capsule* **2013**, *27*, 537–538.
14. Yin, M.; Yang, Z.; Wang, J. 50 Cases of Psoriasis Vulgaris Treated with Zhuhuang Granule No. Ⅱ, Keyin Cream Combined with NB-UVB. *Hunan J. Tradit. Chin. Med.* **2013**, *29*, 56–57, doi:10.16808/j.cnki.issn1003-7705.2013.10.031.
15. Yu, B.; Xie, Y.; Sheng, G. Clinical Study on Tuiyin Decoction Combined with NB-UVB in the Treatment of Psoriasis Vulgaris. *Lishizhen Med. Mater. Medica Res.* **2014**, *25*, 125–127, doi:10.3969 /j.issn.1008-0805.2014.01.053.
16. Zhang, J.; Zhang, W.; Xia, X.; Zhao, G.; Wang, C.; Peng, X.; Lu, G. Efficacy of Narrow-Band Ultraviolet B Combined with Liangxue Xiaofeng Tang in Treating Patients with Psoriasis Vulgaris of Blood-Heat Type. *Chin. J. Dermatovenereology Integr. Tradit. West. Med.* **2014**, *13*, 234–236.
17. Cheng, G. Clinical Observation on Treating Diandi Psorasis with the Qingre Liangxue Decoction plus NB-UVB Rays. *Clin. J. Chin. Med.* **2015**, *7*, 9–10, doi:l0.3969/j.issn.1674-7860.2015.17.004.
18. Hu, Y.; Zhanmg, J.; Dai, S.; Qu, W.; Wang, B. Clinical Analysis of Zidan Yinxie Particles Combined Narrow Band Ultraviolet Treat Psoriasis Vulgaris Disease. *Chin. J. Exp. Tradit. Med. Formulae* **2015**, *21*, 148–151, doi:10.13422/j.cnki.syfjx.2015240148.
19. Pang, L.; Wang, F.; Xue, F. Thirty-Seven Cases with Psoriasis Vulgaris Treated with Wind-Dispersing and Toxin-Eliminating Capsules. *Henan Tradit. Chin. Med.* **2015**, *35*, 3075–3077, doi:10.16367/j.issn.1003-5028.2015.12.1321.
20. Zheng, X.; Liu, H.; Huang, Y.; Chen, H. Curative Effect of Runzao Zhiyang Capsule Combined with UVB in the Treatment of Psoriasis and the Effect to T Cell Subset Level. *China Med. Her.* **2015**, *12*, 124–127.
21. Zou, J.; Chen, G.; Shang, N. Observation Effect of Total Glucosides of Paeony Combination With NBUVB Irradiation Treatment of Plaque Psoriasis. *China Contin. Med. Educ.* **2015**, *7*, 203–205, doi:10.3969/j.issn.1674 9308.2015.13.169.
22. Li, X.; Li, Z.; Li, X. Curative Effect of Total Glucosides of Paeony Capsule Combined with Narrow-Band Ultraviolet B（NB-UVB） in the Treatment of Psoriasis and the Effect to T Cell Subset Level. *Chin. J. Dermatovenereology* **2016**, *30*, 1205–1207, doi:10.13735/j.cjdv.1001-7089.201605118.
23. Liu, X.; Yang, F.; Li, Y. Narrow Band Ultraviolet Combined with Ziyin Liangxue Decoction in the Treatment of Psoriasis Vulgaris. *Shaanxi J. Tradit. Chin. Med.* **2016**, *37*, 596=597, doi:10.3969/j.issn.1000-7369.2016.05.033.

24. Song, H.; Xue, X. Efficacy of Xiaoyin Decoction Combined with Narrow Band Ultraviolet for Psoriasis Vulgaris of Blood-Heat Type and Effect on Expression Levels of Interleukin-17,Interleukin-23 and Interleukin-6 of Peripheral Blood. *Chin. J. Exp. Tradit. Med. Formulae* **2016**, *22*, 192–196, doi:10.13422/j.cnki.syfjx.2016050192.
25. Wu, K. Clinical Observation of Runzaozhiyang Capsule Combined with NB-UVB in Treating 29 Cases of Psoriasis Vulgaris with Blood Deficiency and Wind Dryness. *Chin. J. Dermatovenereology* **2016**, *30*, 1313–1316, doi:10.13735/j.cjdv.1001-7089.201604040.
26. Zhou, Q.; Wang, Z. Clinical Observation of Runzao Zhiyang Capsule Combined with Narrow Band Ultraviolet B in Treating Psoriasis Vulgaris with Blood-Deficiency and Wind-Dryness Syndrome. *Chin. J. Dermatovenereology Integr. Tradit. West. Med.* **2016**, *15*, 174–176.
27. Zhu, X. Effects of Narrow-Band Medium-Wave Ultraviolet Rays Combined with Xiaoyin Granules in the Treatment of Psoriasis on Serum TNF-α, IL-17, and IL-23 Levels in Patients. *Mod. J. Integr. Tradit. Chin. West. Med.* **2016**, *25*, 3375–3377, doi:10.3969/j.issn.1008-8849.2016.30.025.
28. Ji, G. Clinical Study on Zidan Yinxie Capsule Combined with Narrow Band Ultraviolet in Treating Psoriasis Vulgaris of Syndrome of Wind and Dryness Due to Blood Deficiency. *Acta Chin. Med.* **2017**, *32*, 2001–2004, doi:10.16368/j.issn.1674-8999.2017.10.525.
29. Li, L. Observation on the Efficacy of Biejiajian Pills Combined with Western Medicine in the Treatment of Blood-Heat Type Psoriasis Vulgaris. *Chin. Tradit. Pat. Med.* **2017**, *39*, 2213–2215, doi:10.3969/j.issn.1001-1528.2017.10.052.
30. Lin, J. The Efficacy of Total Glycosides of Paeony Capsules Combined with Calcipotriol Ointment in the Treatment of Psoriasis Vulgaris and Its Effect on Serum Inflammatory Factors in Patients. *J. Med. Theory Pract.* **2017**, *30*, 1959–1960, doi:10.19381/j.issn.1001-7585.2017.13.045.
31. Sun, W.; Yan, H.; Wen, Y. Clinical Study of Bai Bi Decoction 2 Combined with NB-UVB in the Treatment of Psoriasis. *Chin. Community Dr.* **2017**, *33*, 90–91.
32. Xie, C.; Fu, M.; Shi, N. The Efficacy of Laser Phototherapy Combined with Compound Qingdai Capsule in the Treatment of Psoriasis and Its Effect on Serum TNF-α, IL-17, and IL-23 Levels. *Mod. J. Integr. Tradit. Chin. West. Med.* **2017**, *26*, 2363–2364, doi:10.3969/j.issn.1008-8849.2017.21.030.
33. Shi, Z. Observation on the Efficacy of Self-Prepared Liangxue Zhiyang Prescription Combined with Medium-Wave Ultraviolet Rays in the Treatment of Psoriasis Vulgaris with Blood-Heat Syndrome in the Acute Stage. *Mod. J. Integr. Tradit. Chin. West. Med.* **2018**, *27*, 966–969, doi:10.3969/j.issn.1008-8849.2018.09.015.
34. Xue, X.; Song, H. Clinical Effects of XiaoYin Decoction Combined with NB-UVB in Treating Psoriasis Vulgaris of Blood-Heat Pattern and the Influence on Th1/Th2 Cytokine in the Serum. *West. J. Tradit. Chin. Med.* **2018**, *31*, 86–88.
35. Mao, C.; Su, M.; Zhou, S.; Yang, R. Clinical Observation of Liangxue Jiedu Decoction Combined with Narrow-Band UVB in the Treatment of Blood Heat Syndrome of Psoriasis Vulgaris. *J. Pract. Dermatol.* **2019**, *12*, 106–108, doi:10.11786/sypfbxzz.1674-1293.20190213.

36. Meng, H. Liangxue Xiaoyin Decoction Combined with Narrow-Band Mid-Wave Ultraviolet Rays in the Treatment of Common Blood-Heat Syndrome Observation on the Efficacy of Psoriasis. *Chin. J. Dermatovenereology Integr. Tradit. West. Med.* **2020**, *19*, 161–163.
37. Liu, X. Observation on the Efficacy of Liangxue Shufeng Decoction Assisted with Narrow-Band Ultraviolet Light Therapy in the Treatment of Psoriasis Vulgaris. *Pract. Clin. J. Integr. Tradit. Chin. West. Med.* **2021**, *21*, 55–56, doi:10.13638/j.issn.1671-4040.2021.23.026.
38. Guo, X.; Zhou, J.; Chen, L. Influence on Huaixiang Wushe Particles with NB-UVB in Treatment of Psoriasis（Blood Heat Syndrome）of Clinical Efficacy and T Cell Subsets. *Chin. J. Dermatovenereology Integr. Tradit. West. Med.* **2021**, *20*, 35–38.
39. Lu, X.; Yang, M.; Wang, H.; Liu, Z. Clinical Observation on the Treatment of Pediatric Psoriasis with Xiaofengsan Combined with Traditional Chinese Medicine Starch Bath and NB-UVB. *J. Shandong Med. Coll.* **2021**, *43*, 291–293, doi:10.3969/j.issn.1674-0947.2021.04.022.
40. Liu, X.; Fu, X.; Zheng, C. Effects of Narrow-Band UVB and Huoxue Jiedu Recipe on Hemorheology and Serum Th1/Th2 Cytokine and IL-17 Expression in Patients with Blood Stasis Psoriasis. *Chin. J. Aesthetic Med.* **2022**, *31*, 102–106, doi:10.15909/j.cnki.cn61-1347/r.004852

Supplementary Materials S3A. Overview of EAHM prescription name and formulation information for each study.

Author	year	Name of formula	Abbreviations	Type of preparation
Zhong	2007	Xiaoyin Granules1	XG1	Granules
Lu	2009	Keyin Pill	KP	Pills
Lu	2010	Investigator Presciption1	IP1	Decoction
Gu	2011	Xiaofeng Granules	XFG	Granules
Liu	2011	Xiaoyin Granules2	XG2	Granules
Pang	2011	Total Glucosides of Paeony Capsules	TGPC	Capsules
Xia	2011	Liangxue Xiaofeng Decoction	LXFD	Decoction
Xu	2011	Investigator Presciption2	IP2	Decoction
Zhang	2011	Liangxue Xiaobian Decoction	LXBD	Decoction
Chen	2012	Qingri Jiedu Huoxue Decoction	QJHD	Decoction
Li	2012	Investigator Presciption3	IP3	Decoction
Han	2013	Investigator Presciption3	IP3	Decoction
Wang	2013	Yinxie Capsule	YXC	Capsules
Yin	2013	Zhuhuang Granules	ZG	Granules
Yu	2014	Tuiyin Decoction	TYD	Decoction
Zhang	2014	Liangxue Xiaofeng Decoction	LXFD	Decoction
Cheng	2015	Qingre Liangxue Decoction	QLD	Decoction
Hu	2015	Zidan Yinxie Granules	ZYD	Granules
Pang	2015	Shufeng Jiedu Capsules	SJC	Capsules
Zheng	2015	Runzao Zhiyang Capsule	RZC	Capsules
Zou	2015	Total Glucosides of Paeony Capsules	TGPC	Capsules
Li	2016	Total Glucosides of Paeony Capsules	TGPC	Capsules
Liu	2016	Ziyin Liangxue Decoction	ZLD	Decoction
Song	2016	Xiaoyin Decoction	XD	Decoction
Wu	2016	Runzao Zhiyang Capsule	RZC	Capsules
Zhou	2016	Runzao Zhiyang Capsule	RZC	Capsules
Zhu	2016	Xiaoyin Granules3	XG3	Granules
Ji	2017	Zidan Yinxie Granules	ZYD	Granules

Li	2017	Biejia Jian Pills	BJP	Pills
Lin	2017	Total Glucosides of Paeony Capsules	TGPC	Capsules
Sun	2017	Bai Bi Decoction	BBD	Decoction
Xie	2017	Compound Qingdai Capsule	CQC	Capsules
Shi	2018	Liangxue Zhiyang Decoction	LZD	Decoction
Xue	2018	Xiaoyin Decoction	XD	Decoction
Mao	2019	Liangxue Jiedu Decoction	LJD	Decoction
Meng	2020	Modified Liangxue Xiaoyin Decoction	MLXD	Decoction
Liu	2021	Liangxue Shufeng Decoction	LSFD	Decoction
Guo	2021	Huaixiang Wushe Granule	HWG	Granules
Lu	2021	Xiaofeng San	XFS	Powders
Liu	2022	Huoxue Jiedu Decoction	HJD	Decoction

Supplementary Materials S3B. Details of the herbs that constitute the EAHM formulation for each study.

Author	year	Abbreviations	Detailed composition of the drug EAHM formulation
Zhong	2007	XG1	*Rehmannia glutinosa* (Gaertn.) DC. Recens, *Angelica sinensis* (Oliv.) Diels, *Paeonia veitchii* Lynch, *Ligusticum striatum* DC., *Arnebia euchroma* I.M.Johnst., *Curcuma phaeocaulis* Valeton, *Scleromitrion diffusum (*Willd.) R.J.Wang, *Smilax glabra* Roxb. Rhizoma, *Prunus mume* Siebold & Zucc., *Glycyrrhiza glabra* L. / Dose information for individual herbs is not reported.
Lu	2009	KP	*Smilax glabra* Roxb. Rhizoma, *Bistorta manshuriensis* Kom., *Menispermum dauricum* DC., *Dictamnus dasycarpus* Turcz. / Dose information for individual herbs is not reported.
Lu	2010	IP1	*Gazella subgutturosa* (Guldenstaedt) 0.9g, *Taraxacum mongolicum* Hand.-Mazz. 15g, *Scleromitrion diffusum* (Willd.) R.J.Wang 12g, *Isatis tinctoria* L. Radix 15g, *Isatis tinctoria* L. Folium 20g, *Scutellaria baicalensis* Georgi 9g, *Imperata cylindrica* (L.) Raeusch. 12g, *Paeonia × suffruticosa* Andrews 9g, *Gardenia jasminoides* J.Ellis 12g, *Dictamnus dasycarpus* Turcz. 12g, *Saposhnikovia divaricata* (Turcz.) Schischk. 12g, *Rehmannia glutinosa* (Gaertn.) DC. Recens 9g, *Bubalus bubalis* Linnaeus 6g, *Glycyrrhiza glabra* L. 9g
Gu	2011	XFG	*Rehmannia glutinosa* (Gaertn.) DC. Recens, *Paeonia × suffruticosa* Andrews, *Arnebia euchroma* I.M.Johnst., *Anemarrhena asphodeloides* Bunge, *Nepeta tenuifolia* Benth., *Saposhnikovia divaricata* (Turcz.) Schischk., *Sophora flavescens* Aiton, *Cryptotympana pustulata* Fabricius, *Glycyrrhiza glabra* L. / Dose information for individual herbs is not reported.
Liu	2011	XG2	*Rehmannia glutinosa* (Gaertn.) DC. Recens, *Paeonia × suffruticosa* Andrews, *Lonicera japonica* Thunb., *Paeonia veitchii* Lynch, *Angelica sinensis* (Oliv.) Diels, *Sophora flavescens* Aiton, *Arctium lappa* L., *Cryptotympana pustulata* Fabricius, *Dictamnus dasycarpus* Turcz., *Isatis tinctoria* L. Folium, *Carthamus tinctorius* L., *Saposhnikovia divaricata (Turcz.)* Schischk., *Scrophularia ningpoensis* Hemsl. / Dose information for individual herbs is not reported.
Pang	2011	TGPC	*Paeonia lactiflora* Pall. / Dose information for individual herbs is not reported.
Xia	2011	LXFD	*Rehmannia glutinosa* (Gaertn.) DC. Recens 30g, Gypsum Fibrosum 30g, *Paeonia lactiflora* Pall. 12g, *Imperata cylindrica* (L.) Raeusch. 30g, *Lonicera japonica* Thunb. 15g, *Nepeta tenuifolia* Benth. 9g, *Saposhnikovia divaricata* (Turcz.) Schischk. 9g, *Scrophularia ningpoensis* Hemsl. 9g, *Anemarrhena asphodeloides* Bunge 9g, *Arctium lappa* L. 9g *Glycyrrhiza glabra* L. 6g, *Actaea heracleifolia* (Kom.) J.Compton 3g
Xu	2011	IP2	*Smilax glabra* Roxb. Rhizoma 30g, *Styphnolobium japonicum* (L.) Schott 15g, *Reynoutria japonica* Houtt. 20g, *Scleromitrion diffusum* (Willd.) R.J.Wang 20g, *Rehmannia glutinosa* (Gaertn.) DC. Recens 10g, *Scrophularia ningpoensis* Hemsl. 10g, *Pericaeta communisma* Gate et Hatai 10g, *Glycyrrhiza glabra* L. 5g
Zhang	2011	LXBD	*Rehmannia glutinosa* (Gaertn.) DC. Recens 20g, *Rehmannia glutinosa* (Gaertn.) DC. Preparata 20g, *Bubalus bubalis* Linnaeus 15g, *Scrophularia ningpoensis* Hemsl. 10g, *Paeonia × suffruticosa* Andrews 10g, *Paeonia veitchii* Lynch 10g, *Imperata cylindrica* (L.) Raeusch. 10g, *Styphnolobium japonicum* (L.) Schott 15g, *Potentilla indica* (Andrews) Th.Wolf 15g, *Scutellaria barbata* D.Don 20g, *Scleromitrion diffusum* (Willd.) R.J.Wang 15g
Chen	2012	QJHD	*Taraxacum mongolicum* Hand.-Mazz. 30g, *Isatis tinctoria* L. Rhizoma 30g, *Paris polyphylla* Sm. 30g, *Scleromitrion diffusum* (Willd.) R.J.Wang 15g, *Sparganium stoloniferum* (Buch.-Ham. ex Graebn.) Buch.-Ham. ex Juz. 15g, *Curcuma phaeocaulis* Valeton 15g, *Paeonia × suffruticosa* Andrews 10g, *Spatholobus suberectus* Dunn 30g, *Bombyx mori* Linné
Li	2012	IP3	*Rehmannia glutinosa* (Gaertn.) DC. Recens 20g, *Scrophularia ningpoensis* Hemsl. 15g, *Ophiopogon japonicus* (Thunb.) Ker Gawl.15g, *Paeonia × suffruticosa* Andrews 10g , *Salvia miltiorrhiza* Bunge 15g, *Angelica sinensis* (Oliv.) Diels 12g, *Polygonatum sibiricum* Redouté 15g, *Smilax glabra* Roxb. Rhizoma 20g, *Astragalus mongholicus* Bunge 20g, *Nepeta tenuifolia* Benth.10g, *Glycyrrhiza glabra* L. 10g
Han	2013	IP3	*Rehmannia glutinosa* (Gaertn.) DC. Recens, *Scrophularia ningpoensis* Hemsl., *Ophiopogon japonicus* (Thunb.) Ker Gawl., *Paeonia × suffruticosa* Andrews, *Salvia miltiorrhiza* Bunge, *Angelica sinensis* (Oliv.) Diels, *Polygonatum sibiricum* Redouté, *Smilax glabra* Roxb. Rhizoma, *Astragalus mongholicus* Bunge, *Nepeta tenuifolia* Benth. *Glycyrrhiza glabra* L. / Dose information for individual herbs is not reported.
Wang	2013	YXC	*Smilax glabra* Roxb. Rhizoma, *Smilax glabra* Roxb. Folium / Dose information for individual herbs is not reported.
Yin	2013	ZG	*Bupleurum chinense* DC., *Paeonia lactiflora* Pall., *Coptis chinensis* Franch., *Scutellaria baicalensis* Georgi, *Phellodendron chinense* var. glabriusculum C.K.Schneid., *Gardenia jasminoides* J.Ellis, *Lophatherum gracile* Brongn., Gypsum Fibrosum, *Ophiopogon japonicus* (Thunb.) Ker Gawl., *Leuzea uniflora* (L.) Holub / Dose information for individual herbs is not reported.
Yu	2014	TYD	*Rehmannia glutinosa* (Gaertn.) DC. Recens 30g, *Smilax glabra* Roxb. Rhizoma 30g, *Angelica sinensis* (Oliv.) Diels 15g, *Reynoutria multiflora* (Thunb.) Moldenke Preparata 15g, *Tribulus terrestris* var. terrestris 15g, *Ligustrum lucidum* W.T.Aiton 10g, *Polygonatum sibiricum* Redouté 10g, *Ophiopogon japonicus* (Thunb.) Ker Gawl. 10g, *Zaocys dhumnades* Cantor 10g, *Scolopendra subspinipes* mutilans Linné Koch 10g, *Lonicera japonica* Thunb. 10g, *Paeonia × suffruticosa* Andrews 10 g, *Prunus persica (*L.) Batsch 10g, *Carthamus tinctorius* L. 10g, *Glycyrrhiza glabra* L. 5g

Zhang	2014	LXFD	*Rehmannia glutinosa* (Gaertn.) DC. Recens, *Scrophularia ningpoensis* Hemsl., *Paeonia lactiflora* Pall., Gypsum Fibrosum, *Anemarrhena asphodeloides* Bunge, *Imperata cylindrica* (L.) Raeusch., *Arctium lappa* L., *Nepeta tenuifolia* Benth., *Saposhnikovia divaricata* (Turcz.) Schischk., *Glycyrrhiza glabra* L., *Actaea heracleifolia* (Kom.) J.Compton, *Lonicera japonica* Thunb. / Dose information for individual herbs is not reported.
Cheng	2015	QLD	*Lonicera japonica* Thunb. 20g, *Forsythia suspensa* (Thunb.) Vahl 15g, *Scutellaria baicalensis* Georgi 12g, *Paeonia × suffruticosa* Andrews 12g, *Paeonia veitchii* Lynch 12g, *Rehmannia glutinosa* (Gaertn.) DC. Recens 12g, *Campsis grandiflora* (Thunb.) K.Schum. Flos 10g, *Arnebia euchroma* I.M.Johnst. 10g, *Glycyrrhiza glabra* L. 9g
Hu	2015	ZYD	Halite Violaceous, *Senna obtusifolia* (L.) H.S.Irwin & Barneby, *Aconitum carmichaeli* Debeaux Preparata, *Zingiber officinale* Roscoe, *Neolitsea cassia* (L.) Kosterm. Ramulus, *Atractylodes macrocephala* Koidz., *Paeonia lactiflora* Pall., *Astragalus mongholicus* Bunge, *Salvia miltiorrhiza* Bunge, *Dalbergia odorifera* T.C.Chen / Dose information for individual herbs is not reported.
Pang	2015	SJC	*Reynoutria japonica* Houtt., *Forsythia suspensa* (Thunb.) Vahl, *Isatis tinctoria* L. Rhizoma, *Bupleurum chinense* DC., *Patrinia scabiosifolia* Link, *Phragmites australis* (Cav.) Trin. ex Steud., *Glycyrrhiza glabra* L. / Dose information for individual herbs is not reported.
Zheng	2015	RZC	*Rehmannia glutinosa* (Gaertn.) DC. Recens, *Reynoutria multiflora* (Thunb.) Moldenke Recens, *Reynoutria multiflora* (Thunb.) Moldenke Preparata, *Sophora flavescens* Aiton, *Salvia miltiorrhiza* Bunge, *Girardinia diversifolia* (Link) Friis, *Morus alba* L. / Dose information for individual herbs is not reported.
Zou	2015	TGPC	*Paeonia lactiflora* Pall. / Dose information for individual herbs is not reported.
Li	2016	TGPC	*Paeonia lactiflora* Pall. / Dose information for individual herbs is not reported.
Liu	2016	ZLD	*Salvia miltiorrhiza* Bunge 30g, *Rehmannia glutinosa* (Gaertn.) DC. Recens 15g, *Rehmannia glutinosa* (Gaertn.) DC. Preparata 15g, *Ophiopogon japonicus* (Thunb.) Ker Gawl.12g, *Angelica sinensis* (Oliv.) Diels 12g, *Paeonia × suffruticosa* Andrews 12g, *Reynoutria multiflora* (Thunb.) Moldenke Preparata 12g, *Scrophularia ningpoensis* Hemsl. 10g, *Paeonia veitchii* Lynch 10g, *Anemarrhena asphodeloides* Bunge 10g, *Zaocys dhumnades* Cantor 10g, *Cryptotympana pustulata* Fabricius 6g, *Glycyrrhiza glabra* L. 6g
Song	2016	XD	*Scutellaria baicalensis* Georgi 10g, *Rehmannia glutinosa* (Gaertn.) DC. Recens 15g, *Paeonia × suffruticosa* Andrews 12g, *Paeonia veitchii* Lynch 15g, *Scutellaria barbata* D.Don 15g, *Scleromitrion diffusum* (Willd.) R.J.Wang 30g, *Styphnolobium japonicum* (L.) Schott 15g, *Arnebia euchroma* I.M.Johnst. 10g, *Scrophularia ningpoensis* Hemsl. 15g, *Salvia miltiorrhiza* Bunge 15g, *Spatholobus suberectus* Dunn 15g, *Glycyrrhiza glabra* L. 6g
Wu	2016	RZC	*Rehmannia glutinosa* (Gaertn.) DC. Recens, *Reynoutria multiflora* (Thunb.) Moldenke Recens, *Reynoutria multiflora* (Thunb.) Moldenke Preparata, *Sophora flavescens* Aiton, *Salvia miltiorrhiza* Bunge, *Girardinia diversifolia* (Link) Friis, *Morus alba* L. / Dose information for individual herbs is not reported.
Zhou	2016	RZC	*Rehmannia glutinosa* (Gaertn.) DC. Recens, *Reynoutria multiflora* (Thunb.) Moldenke Recens, *Reynoutria multiflora* (Thunb.) Moldenke Preparata, *Sophora flavescens* Aiton, *Salvia miltiorrhiza* Bunge, *Girardinia diversifolia* (Link) Friis, *Morus alba* L. / Dose information for individual herbs is not reported.
Zhu	2016	XG3	*Smilax glabra* Roxb. Rhizoma, Hominis Placenta, *Potentilla indica* (Andrews) Th.Wolf, *Isatis tinctoria* L. Folium, *Scleromitrion diffusum* (Willd.) R.J.Wang, *Arnebia euchroma* I.M.Johnst., *Styphnolobium japonicum* (L.) Schott, *Bubalus bubalis* Linnaeus, *Imperata cylindrica* (L.) Raeusch., *Sinomenium acutum* (Thunb.) Rehder & E.H.Wilson, *Trachelospermum jasminoides* (Lindl.) Lem., *Lonicera japonica* Thunb. / Dose information for individual herbs is not reported.
Ji	2017	ZYD	Halite Violaceous, *Senna obtusifolia* (L.) H.S.Irwin & Barneby, *Aconitum carmichaeli* Debeaux Preparata, *Zingiber officinale* Roscoe, *Neolitsea cassia* (L.) Kosterm. Ramulus, *Atractylodes macrocephala* Koidz., *Paeonia lactiflora* Pall., *Astragalus mongholicus* Bunge, *Salvia miltiorrhiza* Bunge, *Dalbergia odorifera* T.C.Chen / Dose information for individual herbs is not reported.
Li	2017	BJP	*Pelodiscus sinensis* Wiegmann 3.6g, *Iris domestica* (L.) Goldblatt & Mabb. 0.9g, *Scutellaria baicalensis* Georgi 0.9g, *Bupleurum chinense* DC. 1.8g, *Armadillidium vulgare* Latreille 0.9g, *Zingiber officinale* Roscoe 0.9g, *Rheum tanguticum* Maxim. ex Balf. 0.9g, *Paeonia lactiflora* Pall. 1.5g, *Neolitsea cassia* (L.) Kosterm. Ramulus 0.9g, *Descurainia sophia* (L.) Webb ex Prantl 0.3g, *Pyrrosia sheareri* (Baker) Ching 0.9g, *Magnolia officinalis* Rehder & E.H.Wilson 0.9g, *Paeonia × suffruticosa* Andrews 1.5g, *Dianthus superbus* L.0.6g, *Campsis grandiflora* (Thunb.) K.Schum. Rhizoma 0.9g,. *Panax ginseng* C.A.Mey. 0.3g, *Eupolyphaga sinensis* Walker 1.5g, *Equus asinus* Linné 1.2g, Vespae Nidus 1.2g, Aphonítrum 3.6g, *Catharsius molossus* Linnaeus 1.8g, *Prunus persica* (L.) Batsch 0.6g
Lin	2017	TGPC	*Paeonia lactiflora* Pall. / Dose information for individual herbs is not reported.
Sun	2017	BBD	*Rehmannia glutinosa* (Gaertn.) DC. Recens, *Ophiopogon japonicus* (Thunb.) Ker Gawl., *Angelica sinensis* (Oliv.) Diels, *Scrophularia ningpoensis* Hemsl., *Styphnolobium japonicum* (L.) Schott, *Isatis tinctoria* L. Folium, *Isatis tinctoria* L. Rhizoma, *Paeonia veitchii* Lynch, *Dictamnus dasycarpus* Turcz., *Smilax glabra* Roxb. Rhizoma, Vespae Nidus / Dose information for individual herbs is not reported.
Xie	2017	CQC	*Portulaca oleracea* L., *Smilax glabra* Roxb. Rhizoma, *Dictamnus dasycarpus* Turcz., *Angelica dahurica (*Hoffm.) Benth. & Hook.f. ex Franch. & Sav. Strobilanthes cusia (Nees) Kuntze, *Arnebia euchroma* I.M.Johnst., *Salvia miltiorrhiza* Bunge, *Taraxacum mongolicum* Hand.-Mazz., *Dryopteris crassirhizoma* Nakai, *Dioscorea spongiosa* J.Q.XiM.Mizuno & W.L.Zhao, *Prunus mume* Siebold & Zucc., *Schisandra chinensis* (Turcz.) Baill., *Crataegus pinnatifida* Bunge, Massa Medicata Fermentata / Dose information for individual herbs is not reported.
Shi	2018	LZD	*Reynoutria japonica* Houtt. 15g, *Smilax glabra* Roxb. Rhizoma 30g, *Dictamnus dasycarpus* Turcz. 10g, *Styphnolobium japonicum* (L.) Schott 15g, *Scleromitrion diffusum* (Willd.) R.J.Wang 15g, *Salvia miltiorrhiza* Bunge 15g,*Sophora tonkinensis* Gagnep. 6g, *Glycyrrhiza glabra* L. 5g

Xue	2018	XD	*Scutellaria baicalensis* Georgi 10g, *Rehmannia glutinosa* (Gaertn.) DC. Recens 15g, *Paeonia × suffruticosa* Andrews 12g, *Paeonia veitchii* Lynch 15g, *Scutellaria barbata* D.Don 30g, *Scleromitrion diffusum* (Willd.) R.J.Wang 15g, *Styphnolobium japonicum* (L.) Schott 10g, *Arnebia euchroma* I.M.Johnst. 15g, *Scrophularia ningpoensis* Hemsl. 15g, *Salvia miltiorrhiza* Bunge 15g, *Spatholobus suberectus* Dunn 15g, *Glycyrrhiza glabra* L. 6g
Mao	2019	LJD	*Styphnolobium japonicum* (L.) Schott 30g, *Rehmannia glutinosa* (Gaertn.) DC. Recens 15g, *Salvia miltiorrhiza* Bunge 15g, *Imperata cylindrica* (L.) Raeusch. 30g, *Arnebia euchroma* I.M.Johnst. 15g, *Paeonia veitchii* Lynch 15g, *Spatholobus suberectus* Dunn 30g, *Lonicera japonica* Thunb. 15g
Meng	2020	MLXD	*Arnebia euchroma* I.M.Johnst. 10g, *Paeonia × suffruticosa* Andrews 10g, *Paeonia veitchii* Lynch 15g, *Rehmannia glutinosa* (Gaertn.) DC. Recens 15g, *Scleromitrion diffusum* (Willd.) R.J.Wang 15g, *Smilax glabra* Roxb. Rhizoma 15g, *Styphnolobium japonicum* (L.) Schott 30g, *Imperata cylindrica* (L.) Raeusch. 30g, *Spatholobus suberectus* Dunn 15g, *Glycyrrhiza glabra* L. 6g
Liu	2021	LSFD	*Bubalus bubalis* Linnaeus 30g, *Lonicera japonica* Thunb. 15g, *Rehmannia glutinosa* (Gaertn.) DC. Recens 15g, *Isatis tinctoria* L. Folium 15g, *Styphnolobium japonicum* (L.) Schott 15g, *Paeonia × suffruticosa* Andrews 15g, *Paeonia veitchii* Lynch 15g, *Scutellaria baicalensis* Georgi 15g, *Isatis tinctoria* L. 30g
Guo	2021	HWG	*Styphnolobium japonicum* (L.) Schott, *Imperata cylindrica* (L.) Raeusch., *Rehmannia glutinosa* (Gaertn.) DC. Recens, *Arnebia euchroma* I.M.Johnst., *Salvia miltiorrhiza* Bunge, *Spatholobus suberectus* Dunn, *Zaocys dhumnades* Cantor, *Saposhnikovia* divaricata (Turcz.) Schischk. / Dose information for individual herbs is not reported.
Lu	2021	XFS	*Nepeta tenuifolia* Benth.10g, *Saposhnikovia divaricata* (Turcz.) Schischk. 10g, *Sesamum indicum* L. 10g, *Arctium lappa* L. 10g, *Angelica sinensis* (Oliv.) Diels 10g, *Cryptotympana pustulata* Fabricius 12g, *Sophora flavescens* Aiton 12g, *Anemarrhena asphodeloides* Bunge 12g, *Rehmannia glutinosa* (Gaertn.) DC. Recens 12g, *Tetrapanax papyrifer* (Hook.) K.Koch 12g, Gypsum Fibrosum 15g, *Lonicera japonica* Thunb. 15g, *Forsythia suspensa* (Thunb.) Vahl 15g, *Atractylodes lancea* (Thunb.) DC. 9g, *Glycyrrhiza glabra* L. 6g
Liu	2022	HJD	*Scleromitrion diffusum* (Willd.) R.J.Wang 30g, *Spatholobus suberectus* Dunn 30g, *Salvia miltiorrhiza* Bunge 20g, *Scrophularia ningpoensis* Hemsl. 15g, *Curcuma phaeocaulis* Valeton 12g, *Carthamus tinctorius* L. 10g, *Prunus persica* (L.) Batsch 10g, *Euonymus alatus* (Thunb.) Siebold 10g, *Citrus aurantium* L. 9g

All individual herb names are listed according to the accepted name of the international standard plant species database, World Flora Online (WFO, https://www.worldfloraonline.org/). In the case of non-plant material, it is labelled according to the species name of the originating organism as listed in the Korean Pharmacopoeia.

Supplementary Materials S4. (A) Forest plot for Gastrointestinal AE in pairwise meta-analysis; (B) Forest plot for Cutaneous AE in pairwise meta-analysis.

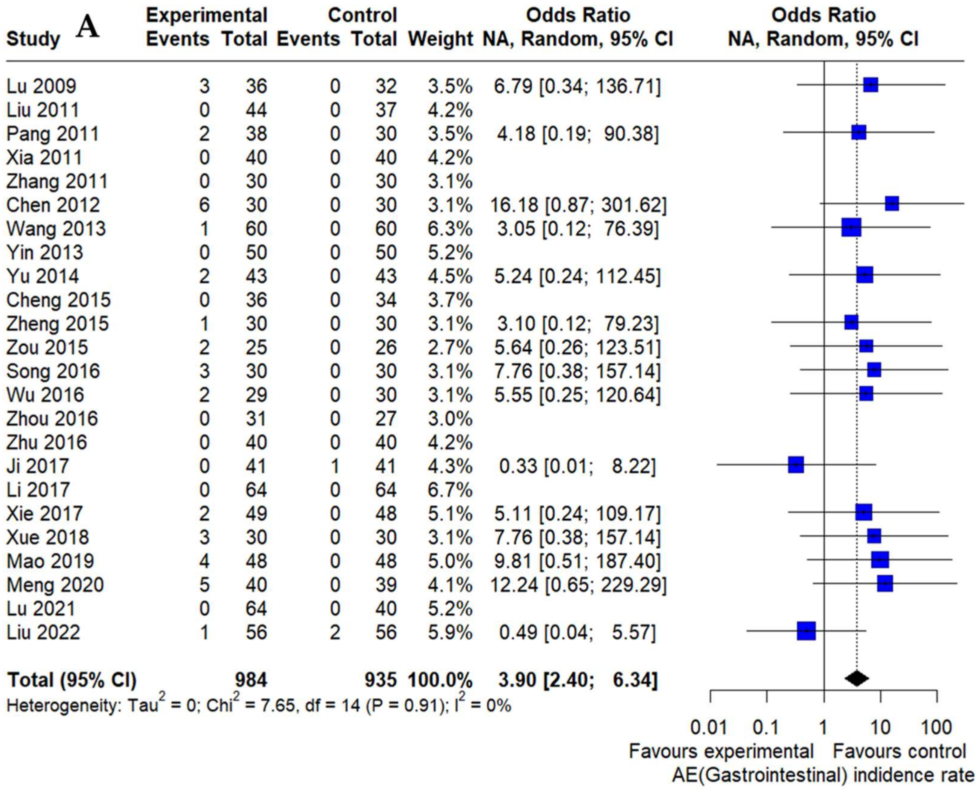

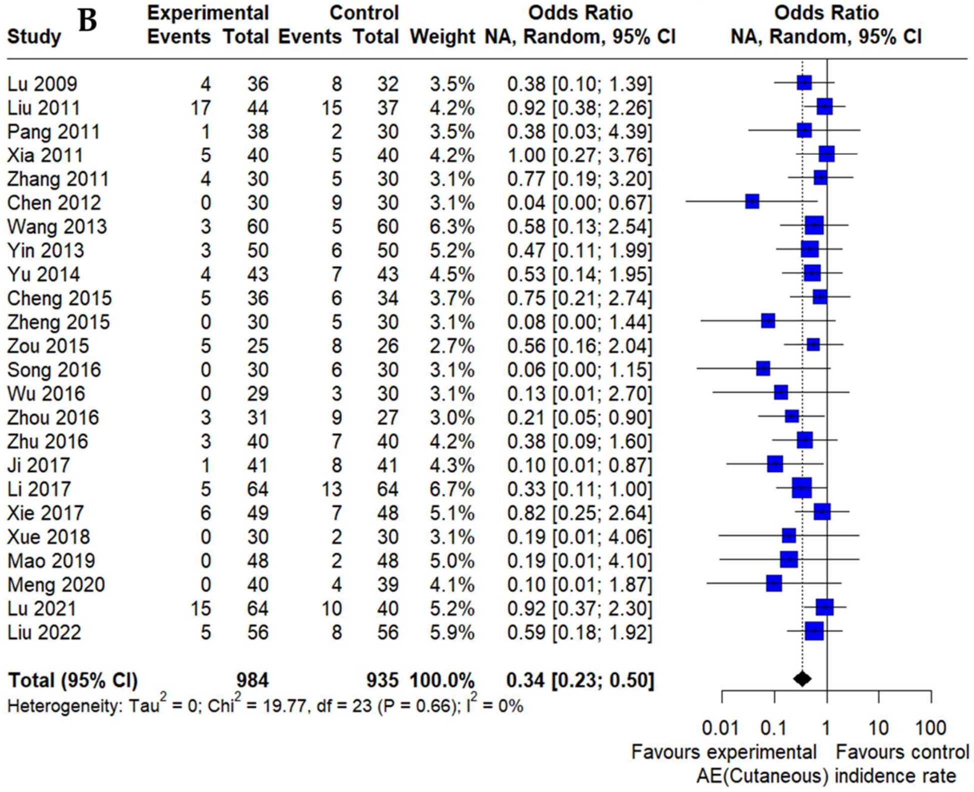

Supplementary Materials S5. PageRank centrality values and rankings of all EAHM prescription herbs included in this study.

Herb name	Rank	PageRank centrality
Paeonia suffruticosa Andrews	1	0.025625
Smilax glabra Roxb. Rhizoma	2	0.023451
Salvia miltiorrhiza Bunge	3	0.023228
Glycyrrhiza glabra L.	4	0.022877
Rehmannia glutinosa (Gaertn.) DC. Recens	5	0.022842
Scutellaria baicalensis Georgi	6	0.019682
Paeonia lactiflora Pall.	7	0.018824
Lonicera japonica Thunb.	8	0.01814
Dictamnus dasycarpus Turcz.	9	0.017707
Arnebia euchroma I.M.Johnst.	10	0.017662
Scleromitrion diffusum (Willd.) R.J.Wang	11	0.017507
Scrophularia ningpoensis Hemsl.	12	0.017414
Prunus persica (L.) Batsch	13	0.016551
Angelica sinensis (Oliv.) Diels	14	0.016184
Ophiopogon japonicus (Thunb.) Ker Gawl.	15	0.014929
Paeonia veitchii Lynch	16	0.014611
Bupleurum chinense DC.	17	0.014589
Saposhnikovia divaricata (Turcz.) Schischk.	18	0.014156
Imperata cylindrica (L.) Raeusch.	19	0.013987
Isatis tinctoria L.	20	0.013413
Taraxacum mongolicum Hand.-Mazz.	21	0.013407
Styphnolobium japonicum (L.) Schott	22	0.013057
Vespae Nidus	23	0.012581
Isatis tinctoria L. Folium	24	0.012414
Neolitsea cassia (L.) Kosterm. Ramulus	25	0.012253
Zingiber officinale Roscoe	26	0.012253
Carthamus tinctorius L.	27	0.011794
Spatholobus suberectus Dunn	28	0.011347
Gypsum Fibrosum	29	0.011299
Sophora flavescens Aiton	30	0.010897
Anemarrhena asphodeloides Bunge	31	0.010837
Cryptotympana pustulata Fabricius	32	0.010809
Bubalus bubalis Linnaeus	33	0.010675
Forsythia suspensa (Thunb.) Vahl	34	0.010576
Nepeta tenuifolia Benth.	35	0.010558
Zaocys dhumnades Cantor	36	0.010436
Reynoutria multiflora (Thunb.) Moldenke Preparata	37	0.010188
Curcuma phaeocaulis Valeton	38	0.010046
Arctium lappa L.	39	0.00974

Prunus mume Siebold & Zucc.	40	0.009446
Gardenia jasminoides J.Ellis	41	0.009428
Dianthus superbus L.	42	0.008985
Iris domestica (L.) Goldblatt & Mabb.	43	0.008985
Pelodiscus sinensis Wiegmann	44	0.008985
Aphonitrum	45	0.008985
Armadillidium vulgare Latreille	46	0.008985
Campsis grandiflora (Thunb.) K.Schum. Rhizoma	47	0.008985
Catharsius molossus Linnaeus	48	0.008985
Descurainia sophia (L.) Webb ex Prantl	49	0.008985
Eupolyphaga sinensis Walker	50	0.008985
Pyrrosia sheareri (Baker) Ching	51	0.008985
Rheum tanguticum Maxim. ex Balf.	52	0.008985
Equus asinus Linn	53	0.008985
Magnolia officinalis Rehder & E.H.Wilson	54	0.008985
Panax ginseng C.A.Mey.	55	0.008985
Astragalus mongholicus Bunge	56	0.008594
Polygonatum sibiricum Redout	57	0.007976
Rehmannia glutinosa (Gaertn.) DC. Preparata	58	0.007851
Potentilla indica (Andrews) Th.Wolf	59	0.007716
Reynoutria japonica Houtt.	60	0.007498
Dryopteris crassirhizoma Nakai	61	0.006823
Strobilanthes cusia (Nees) Kuntze	62	0.006823
Angelica dahurica (Hoffm.) Benth. & Hook.f. ex Franch. & Sav.	63	0.006823
Massa Medicata Fermentata	64	0.006823
Crataegus pinnatifida Bunge	65	0.006823
Portulaca oleracea L.	66	0.006823
Schisandra chinensis (Turcz.) Baill.	67	0.006823
Dioscorea spongiosa J.Q.Xi, M.Mizuno & W.L.Zhao	68	0.006823
Scutellaria barbata D.Don	69	0.006772
Scolopendra subspinipes mutilans Linn Koch	70	0.006484
Tribulus terrestris var. terrestris	71	0.006484
Ligustrum lucidum W.T.Aiton	72	0.006484
Atractylodes lancea (Thunb.) DC.	73	0.006481
Sesamum indicum L.	74	0.006481
Tetrapanax papyrifer (Hook.) K.Koch	75	0.006481
Gazella subgutturosa (Guldenstaedt)	76	0.006076
Hominis Placenta	77	0.005548
Trachelospermum jasminoides (Lindl.) Lem.	78	0.005548
Sinomenium acutum (Thunb.) Rehder & E.H.Wilson	79	0.005548
Atractylodes macrocephala Koidz.	80	0.005303
Aconitum carmichaeli Debeaux Preparata	81	0.005303
Dalbergia odorifera T.C.Chen	82	0.005303

Halite Violaceous	83	0.005303
Senna obtusifolia (L.) H.S.Irwin & Barneby	84	0.005303
Actaea heracleifolia (Kom.) J.Compton	85	0.005301
Leuzea uniflora (L.) Holub	86	0.005015
Coptis chinensis Franch.	87	0.005015
Lophatherum gracile Brongn.	88	0.005015
Phellodendron chinense var. glabriusculum C.K.Schneid.	89	0.005015
Ligusticum striatum DC.	90	0.004712
Bombyx mori Linn	91	0.004614
Paris polyphylla Sm.	92	0.004614
Sparganium stoloniferum (Buch.-Ham. ex Graebn.) Buch.-Ham. ex Juz.	93	0.004614
Citrus aurantium L.	94	0.004433
Euonymus alatus (Thunb.) Siebold	95	0.004433
Campsis grandiflora (Thunb.) K.Schum. Flos	96	0.004219
Sophora tonkinensis Gagnep.	97	0.004054
Pericaeta communisma Gate et Hatai	98	0.004003
Phragmites australis (Cav.) Trin. ex Steud.	99	0.003854
Patrinia scabiosifolia Link	100	0.003854
Girardinia diversifolia (Link) Friis	101	0.003479
Morus alba L.	102	0.003479
Bistorta manshuriensis Kom.	103	0.003064
Menispermum dauricum DC.	104	0.003064
Smilax glabra Roxb. Folium	105	0.001819

All individual herb names are listed according to the accepted name of the international standard plant species database, World Flora Online (WFO, https://www.worldfloraonline.org/). In the case of non-plant material, it is labelled according to the species name of the originating organism as listed in the Korean Pharmacopoeia.
